# Nanococktail Based on AIEgens and Semiconducting Polymers: A Single Laser Excited Image-Guided Dual Photothermal Therapy

**DOI:** 10.7150/thno.41317

**Published:** 2020-01-12

**Authors:** Zi Long, Jun Dai, Qinyu Hu, Quan Wang, Shijie Zhen, Zujin Zhao, Zitong Liu, Jing-Jing Hu, Xiaoding Lou, Fan Xia

**Affiliations:** 1 Engineering Research Center of Nano-Geomaterials of Ministry of Education, Faculty of Materials Science and Chemistry, China University of Geosciences, Wuhan 430074, China.; 2 Department of Obstetrics and Gynecology, Tongji Hospital, Tongji Medical College, Huazhong University of Science and Technology, Wuhan 430074, China.; 3 Beijing National Laboratory for Molecular Sciences, Organic Solids Laboratory, Institute of Chemistry, Chinese Academy of Sciences, Beijing 100190, China.; 4 State Key Laboratory of Luminescent Materials and Devices, Center for Aggregation-Induced Emission, South China University of Technology, Guangzhou 510640, China.

**Keywords:** A single laser, semiconducting polymers, aggregation-induced emission fluorogens, fluorescence resonance energy transfer, dual photothermal therapy

## Abstract

Semiconducting polymers (SPs)-based dual photothermal therapy (PTT) obtained better therapeutic effect than single PTT due to its higher photothermal conversion efficiency. However, most dual PTT need to use two lasers for heat generation, which brings about inconvenience and limitation to the experimental operations. Herein, we report the development of “nanococktail” nanomaterials (DTPR) with 808 nm-activated image-guided dual photothermal properties for optimized cancer therapy.

**Methods**: In this work, we co-encapsulated AIEgens (TPA-BDTO, T) and SPs (PDPPP, P) by using maleimide terminated amphiphilic polymer (DSPE-PEG_2000_-Mal, D), then further conjugated the targeting ligands (RGD, R) through “click” reaction. Finally, such dual PTT nanococktail (termed as DTPR) was constructed.

**Results**: Once DTPR upon irradiation with 808 nm laser, near-infrared fluorescence from T could be partially converted into thermal energy through fluorescence resonance energy transfer (FRET) between T and P, coupling with the original heat energy generated by the photothermal agent P itself, thus resulting in image-guided dual PTT. The photothermal conversion efficiency of DTPR reached 60.3% (dual PTT), much higher as compared to its inherent photothermal effect of only 31.5% (single PTT), which was further proved by the more severe photothermal ablation *in vitro* and *in vivo* upon 808 nm laser irradiation.

**Conclusion**: Such smart “nanococktail” nanomaterials could be recognized as a promising photothermal nanotheranostics for image-guided cancer treatment.

## Introduction

Photothermal therapy (PTT) that converts light into heat to induce cancer-cell death, has arisen as a prospective strategy for cancer treatment owing to its noninvasiveness 1-12. So far, numerous photothermal agents with strong near-infrared (NIR) absorbance have been developed for cancer therapy including silver nanoparticles 13, 14, carbon-based materials (carbon nanotube and graphene oxide) 15-17, gold nanorods 18-20, Pd sheets 21, 22, CuS particles 23, 24, semiconducting polymers (SPs) 25-27, organic NIR dyes 28, 29 and so on 30, 31. Among them, SPs have drawn extensive attention in biomedical fields because of the favourable advantages such as tunable structure, intriguing optical characteristics, ease of processing into nanoparticles and admirable biocompatibility 32-34. Therefore, SPs have sprung up as promising photothermal agents for PTT 35-40. Particularly, SPs-based imaging assisted PTT could not only achieve accurate tumor location, but also obtain the feedback of therapeutic effect. Fan et al. have developed a SP nanotheranostics for NIR II fluorescence/photoacoustic imaging-guided PTT, which the tumor inhibition rate was up to 77% 41. Pu's group designed a novel SP nanococktail for afterglow-imaging guided PTT, the photothermal conversion efficiency of 35% made it an effective therapeutic method 42.

Despite the fact that recent advances on SPs-based imaging assisted PTT have obtained positive results, such reported SPs usually conduct single PTT (Here we refer to the photothermal reagent itself that converts light into heat called single PTT, while the heat transferred in other ways, such as fluorescence resonance energy transfer (FRET), together with the heat generated by the photothermal agents itself called dual PTT). Compared to dual PTT, the photothermal conversion efficiency and therapeutic effect of photothermal agents for single PTT required to be improved. Although few researchers have reported the SPs with dual-peak absorption for dual PTT 43, 44, the two lasers based sequential excitation would make the therapy process complicated. FRET is an energy conversion process from a donor to an acceptor via photo-irradiation. Its efficiency is closely related to the distance and spectral overlap between the donor fluorogens and nearby acceptor molecules 45, 46. As a proof-of-concept, if SPs are rationally combined with FRET, one laser based dual PTT may be achieved. On account of this, it is a strategy to address the issues by simultaneously encapsulating fluorogens and SPs to realize the conversion of partial energy from fluorogens into additional thermal energy from SPs through FRET effect while fluorescence image-guided. To conquer the aggregation caused quenching (ACQ) effect of such encapsulated fluorogens 47, 48, aggregation-induced emission fluorogens (AIEgens) with distinctive AIE property (non-emissive in solution state but strongly emissive in aggregated state) thus can be utilized as ideal fluorogen donors 49-55.

**Scheme 1:**
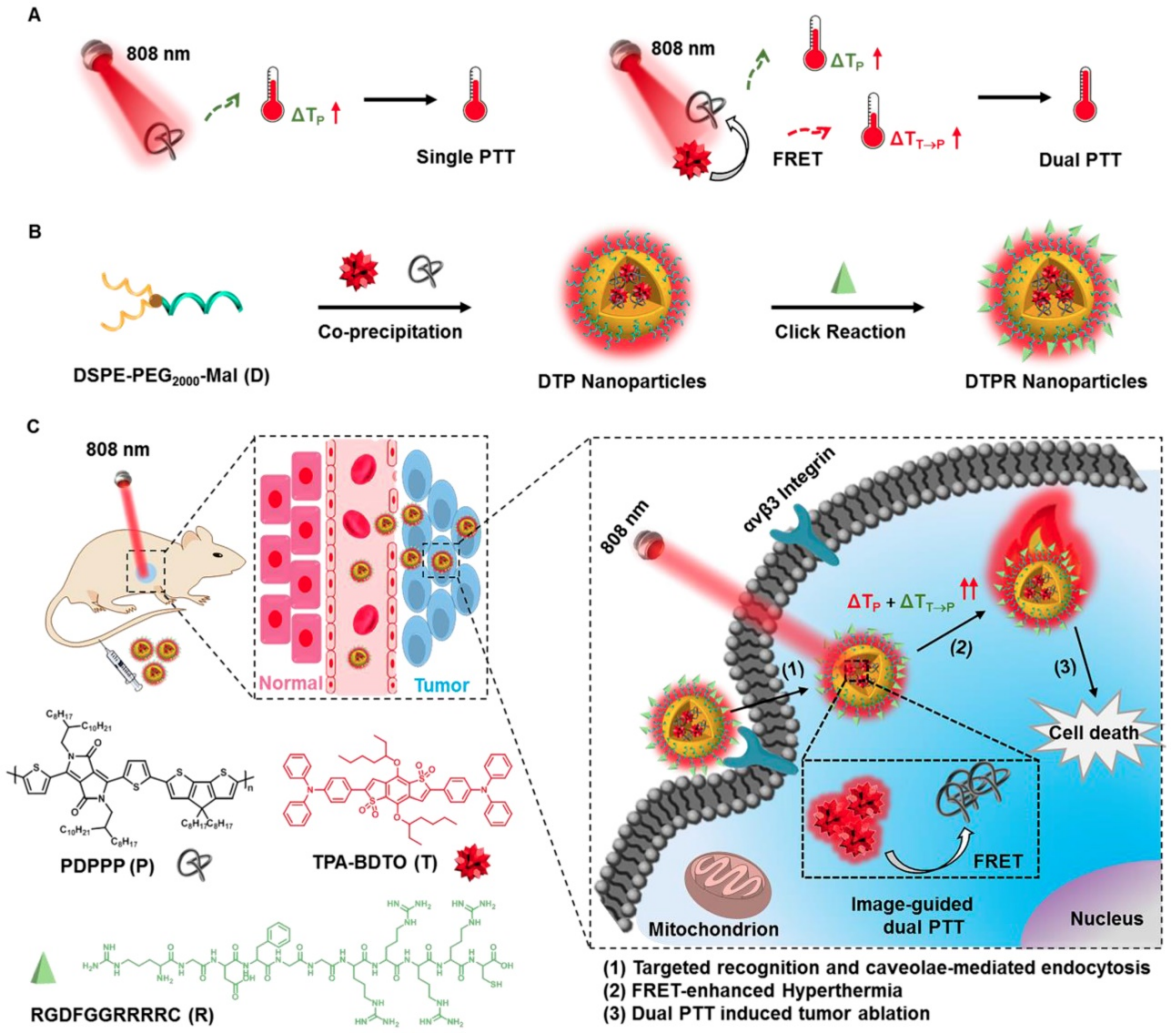
** (A)** Schematic illustration of single and dual PTT strategy under 808 nm laser irradiation. **(B)** Preparation of DTPR nanoparticles. **(C)** Schematic design of DTPR nanoparticles for 808 nm-activated image-guided dual PTT.

Herein, we co-encapsulated AIEgens (TPA-BDTO, T) and SPs (PDPPP, P) by using maleimide terminated amphiphilic polymer (DSPE-PEG_2000_-Mal, D), then further conjugated the targeting ligands (RGD, R) through “click” reaction. Finally, such dual PTT nanococktail (termed as DTPR) was constructed. When excited by 808 nm laser, AIEgens T would exhibit bright red fluorescence, simultaneously transfered partial energy into SPs P via FRET effect to generate additional heat, together with the intrinsic heat of P (**Scheme S1**), thus a single laser excited image-guided dual PTT could be realized (**Scheme 1**). This novel theranostic nanoplatform was designed to contain three parts: (1) a bright red emissive AIEgen T with two-photon absorption property, was not only partially utilized for two-photon fluorescence imaging but also partially acted as the fluorescence resonance energy donor; (2) the RGD peptide with high binding affinity toward αvβ3 integrin receptor, was applied to promote cellular internalization and precision treatment; (3) the SPs P with broad NIR absorption was selected as the photothermal agent and fluorescence resonance energy receptor. *In vitro* and *in vivo* experiments uncovered that DTPR nanoparticles could spark severe cell damage, thus triggered dual photothermal efficacy with serious tumor ablation. We expected that this successful demonstration of multifunctional nanoparticles with image-guided dual PTT characteristics would open a new avenue for SPs nanomaterials in anti-cancer applications.

## Materials and Methods

### Synthesis of DTPR Nanoparticles

DTPR was prepared by a nanoprecipitation method 56-59. T was synthesized on the basis of our former report 60, while P was prepared by the reported literature 61. Briefly, 1 mL THF solution containing 0.5 mg T, P (from 0 to 1 mg/mL, according to the doping amount), and DSPE-PEG_2000_-Mal (2 mg) were quickly injected into 9 mL DI water under continuous sonication at a power output of 300 W for 40 min. After evaporating THF under argon atmosphere, the aqueous solution was filtered via a polythersulfone (PES) syringe-driven filter (0.2 μm) (Millipore), and washed about 3-6 times with a 50 K centrifugal filter units (Millipore) under centrifugation at 5000 r.p.m. for 20 min 59, 62, 63. Thus obtained DTP solution was concentrated to 1 mL by ultrafiltration and stored at 4 °C for further use. For covalently binding RGD to the surface of DTP, a certain amount of SH-RGD (dissolved in DMSO) was added into 0.5 mL aqueous suspension of DTP nanoparticles (molar ratio of DSPE-PEG_2000_-Mal and SH-RGD was 1:3). After the solution was oscillated for 36 h at 37 °C, dialysis (cutoff Mw 3500) against DI water was performed for 72 h to remove unreacted SH-RGD and DMSO. The final obtained suspension of DTPR nanoparticles was filtered by a 0.2 μm filter and stored at 4 °C for further use. The DR, DTR, DPR nanoparticles were prepared in a similar way, see **Table S1** (**Supporting Information**) for details.

## Results and Discussion

### Characterization of Multifunctional DTPR Nanoparticles

AIEgens T was prepared according to our previous report 60, while SPs, P (M_n_ = 50033, polydispersity index (PDI) = 1.4, **Figure S1**) was synthesized according to the reported procedure 61. Initially, we investigated the optical properties of T and P. T was selected as the fluorescence emitter. Its maximum absorption peak and maximum emission peak were located at 530 nm and 660 nm, respectively (**Figure S2A**). It have been reported that T showed good two-photon absorption property, which was believed to be an ideal NIR fluorescence imaging reagent for construction of nanotheranostics 64. Meanwhile, P displayed a broad NIR absorption from 600 to 900 nm with almost no detectable fluorescence emission signal, which favored PTT (**Figure S2B**). By virtue of this, the nanoparticles were fabricated via nano-coprecipitation method using SPs P, AIEgens T and biocompatible block lipid-PEG co-polymer D with maleimide terminated. The optimum doping amount of P : T was 160 w/w %, in which the DTP nanoparticles obtained the highest amount of P but maintained the morphological stability (**Figure S3A, S3C, S4**). Interestingly, the fluorescence of DTP nanoparticles decreased with the increasing doping amount of P, which might be attributed to FRET effect (**Figure S3B**). Taking advantage of the optimal doping amount, we prepared D, DT and DP nanoparticles as control groups (**Table S1**). D, DT, DP, DTP nanoparticles have desirable size and good water dispersibility (**Figure S5A**). DTP showed two absorption peaks where located at 530 nm and 840 nm, arising from T and P, respectively (**Figure S5B**). Both the fluorescence spectra of DTP and DT ranged from 550 to 850 nm with a maximum peak of 660 nm. However, due to the FRET effect, the fluorescence intensity of DTP was weaker than DT nanoparticles under the same conditions (**Figure S5C**).

**Figure 1:**
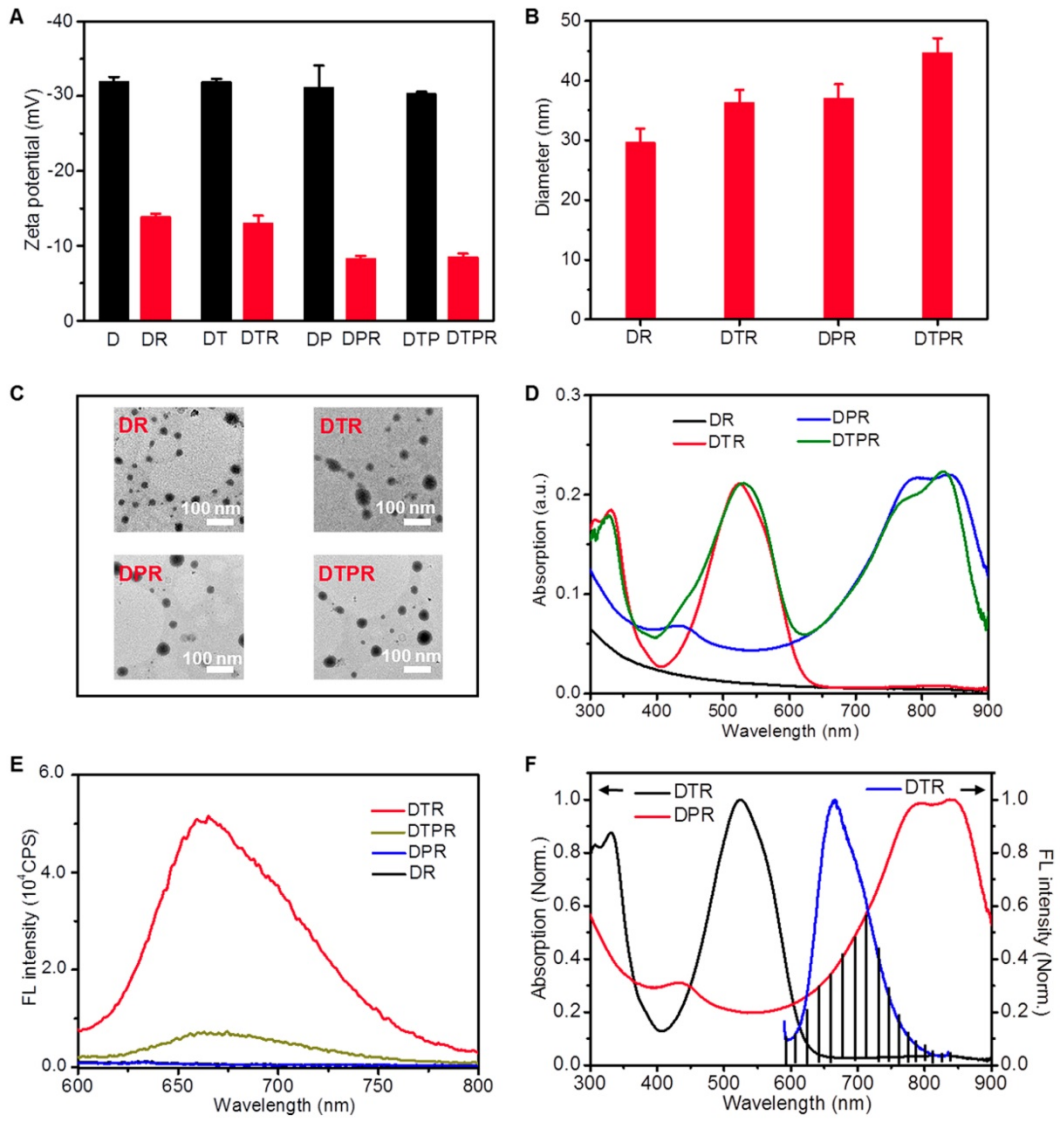
** (A)** Zeta potential change of D, DT, DP, DTP nanoparticles in aqueous solution before and after modification with RGD. **(B)** DLS size distribution of DR, DTR, DPR, DTPR nanoparticles. **(C)** TEM images of DR, DTR, DPR, DTPR nanoparticles in aqueous solution, Scale bar: 100 nm. Absorption **(D)** and FL spectra **(E)** of DR, DTR, DPR, DTPR nanoparticles in aqueous solution, λ_ex_ = 808 nm. **(F)** The normalized absorption and FL spectra of DTR and DPR nanoparticles. Spectra overlay was marked as shadow and represented the mechanism of FRET, λ_ex_ = 808 nm.

To improve the targeting ability to SKOV-3 cells, DTP was further modified with RGD peptide, which had high affinity to αvβ3 integrin that was overexpressed in SKOV-3 cells 65, 66. The zeta potential results showed the success of modification (**Figure 1A**). The actual loading contents of T and P in DTPR were calculated to be 66.0 wt % and 74.4 wt % according to the standard curve, respectively (**Figure S6**). Obviously, the obtained DTPR exhibited similar particle size, absorption and fluorescence spectra, indicating that the conjugation of SH-RGD did not influence the above properties (**Figure 1B, 1C, 1D, 1E, Figure S7**). Transmission electron microscope (TEM) images revealed that DTPR nanoparticles were spherical with an average diameter of about 44 nm, which was in consistent with that of dynamic light scattering (DLS) results (45 nm). Even after some days of storage, the sizes of DR, DTR, DPR, DTPR were hardly changed, demonstrating the attractive stability in water and water containing 10% FBS medium (**Figure S8A, S8B**). Moreover, no matter whether DTPR was stored at room temperature or irradiated by 808 nm laser for a period of time, it could still maintain relatively good fluorescence, indicating that DTPR possessed good photostability (**Figure S8C, S8D**). The emission spectrum of DTR partially overlapped with the absorption spectrum of DPR (shaded section), coinciding with the mechanism of FRET, which was the reason for the decrement of fluorescence (**Figure 1F, Figure S9**). It also further proved that we successfully constructed an efficient FRET system, where T and P served as a donor-acceptor pair.

### FRET-Mediated Photothermal Effect of DTPR Nanoparticles

**Figure 2:**
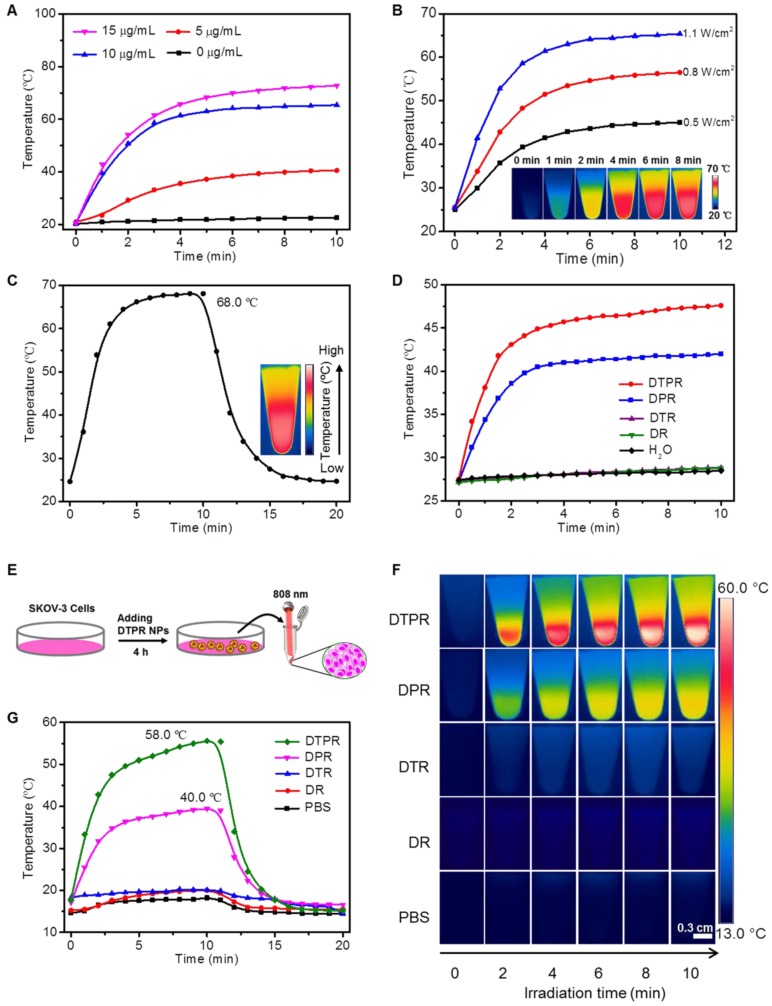
** (A)** Photothermal conversion behavior of DTPR nanoparticles with different concentrations (0, 5, 10 and 15 μg/mL) under 808 nm laser irradiation (1.1 W/cm^2^). **(B)** Photothermal heating curves of aqueous suspensions of dispersed DTPR nanoparticles under irradiation of 808 nm laser at varied power densities (0.5, 0.8 and 1.1 W/cm^2^). Inset: infrared thermal images of 10 μg/mL DTPR nanoparticles. **(C)** Photothermal heating and cooling curves of DTPR nanoparticles (10 μg/mL) under 808 nm laser irradiation (1.1 W/cm^2^). Inset: infrared thermal image of DTPR at its maximum temperature. **(D)** Photothermal effect of H_2_O, DR, DTR, DPR and DTPR (10 μg/mL) under irradiation with a 660 nm laser for 10 min (1.1 W/cm^2^). **(E)** The experimental scheme of SKOV-3 cells after incubated with different nanoparticles (40 μg/mL) for 4 h, then collected and irradiated by 808 nm laser for 10 min. **(F)** Infrared thermal images of SKOV-3 cells under 808 nm laser irradiation (1.1 W/cm^2^) after incubated with different nanoparticles and PBS, respectively. **(G)** Profiles of the temperature increases in SKOV-3 cells shown in **(F)**. Scale bars: 0.3 cm.

To investigate whether DTPR nanoparticles possessed photothermal activities, the following experiments were conducted. Firstly, DTPR of various concentrations (0, 5, 10, 15 and 20 μg/mL) were irradiated by 808 nm laser (1.1 W/cm^2^) to explore the photothermal performance. As depicted in **Figure 2A**, the temperature of DTPR gradually increased with the time and reached the plateau at a concentration of 10 μg/mL. Then, DTPR was exposed to an 808 nm laser with different power densities (0.5, 0.8 and 1.1 W/cm^2^). The temperature of DTPR boosted as the laser intensity increased and reached the maximum at 1.1 W/cm^2^ (**Figure 2B**). The concentration of 10 μg/mL and laser intensity of 1.1 W/cm^2^ were chosed as the optimal conditions for photothermal research in solution. Secondly, as a proof of concept, DTPR could acquire additional heat from T to P via FRET effect coupling with the intrinsic thermal energy from P, thus the maximum heat energy could be obtained. Therefore, we estimated the photothermal effects of DPR and DTPR nanoparticles under the same conditions so as to validate the FRET of DTPR in aqueous solutions. Our results showed that DTPR exhibited much higher increase of solution temperature compared with DPR and reached the highest value of 68.0 °C (t = 10 min), while the maximum temperature of the DPR was only about 48.6 °C (**Figure 2C, Figure S10A**). However, in the control groups, negligible change of temperature was observed in DR and DTR samples (**Figure S10B, 10C**). Due to the FRET effect caused by the partial overlap of the emission spectrum of T with the absorption spectrum of P, we further specifically evaluated whether the 660 nm laser could excite DPR and DTPR to generate heat. **Figure 2D** revealed that DTPR had higher heat generation efficiency than DPR, whereas there were no significant differences in temperature change of H_2_O, DR and DTR. In a result, DTPR combining with FRET effect produced a dual photothermal effect with greater potential to kill cancer cells. Photothermal conversion efficiency was an important parameter to evaluate this application, which was calculated according to the Roper's method 67, 68. The photothermal conversion efficiency of DTPR was 60.3%, which was 1.9-fold higher than DPR (31.5%) and highly correlated with its FRET status (**Figure S11**). These results illustrated that DTPR nanoparticles could quickly trigger dual heat effect under 808 nm laser irradiation, and thus obtain the intensified hyperthermia (~68.0 °C). Furthermore, the photothermal stability in solution was evaluated. The reversible heating-cooling operation disclosed that the maximum temperature of DTPR remained almost unchanged even after seven cycles, indicating good photothermal stability (**Figure S12**). Considering that DTPR had such an excellent photothermal effect in solution, we intended to explore its photothermal effect in living cells. As revealed in **Figure 2E**, after incubated with PBS, DR, DTR, DPR and DTPR nanoparticles for 4 h, respectively, SKOV-3 cells were trypsinized and transferred to a 1.5 ML EP tube then in situ irradiated with 808 nm laser for 10 min to record the photothermal signals. As expected, only P-loaded nanoparticles (DPR and DTPR) displayed photothermal effect (**Figure 2F**). FRET effect elevated the maximum temperature of DTPR to 58.0 °C, while DPR could only rise to 40.0 °C under the same conditions (**Figure 2G**). These results indicated that DTPR caused dual heat in living cells due to the FRET effect.

Meanwhile, we wondered whether 808 nm laser irradiation on DR, DPR, DTR and DTPR could produce reactive oxygen species (ROS) for photodynamic therapy (PDT). Therefore, ABDA was utilized as an indicator to survey the ROS generation ability of nanoparticles. The results showed that DR, DPR, DTR and DTPR could not produce ROS and the 808 nm laser simply excitated DPR, DTPR to produce heat (**Figure S13**).

### *In vitro* Studies of DTPR Nanoparticles

#### RGD Targeting and Endocytosis Mechanism

The targeting peptide RGD could specifically bind to αvβ3 integrin, therefore, the αvβ3 integrin over-expression SKOV-3 cells were chosen for the following experiments 69, 70. The cellular uptake and intracellular localization were observed through confocal laser scanning microscopy (CLSM). The SKOV-3 cells were incubated with different concentrations of DTPR for different time. When excited with two photon (808 nm), significant red fluorescence of DTPR was observed in SKOV-3 cells, yet hardly showed any overlap with blue fluorescence of the nucleus dye Hochest 33258 (**Figure S14, S15, S16**). The Pearson's colocalization coefficient of DTPR and Hochest 33258 was as low as 0.4 (**Figure S17**), illustrating that DTPR was localized in cytoplasm rather than the nucleus. Additionally, the red fluorescence excitated by two-photon (850, 808 nm) producing much higher than one-photon (488 nm, **Figure S18, S29, S20, S21, S22**). Excitingly, similar red fluorescence was also found at the cytoplasm in αvβ3 integrin over-expressed MDA-MB-231, PC3 cells, suggesting the universal cell targeting ability of RGD peptide (**Figure S23**) 53, 71. These results verified that DTPR was a promising two-photon probe for targeting cancer cell imaging.

**Figure 3:**
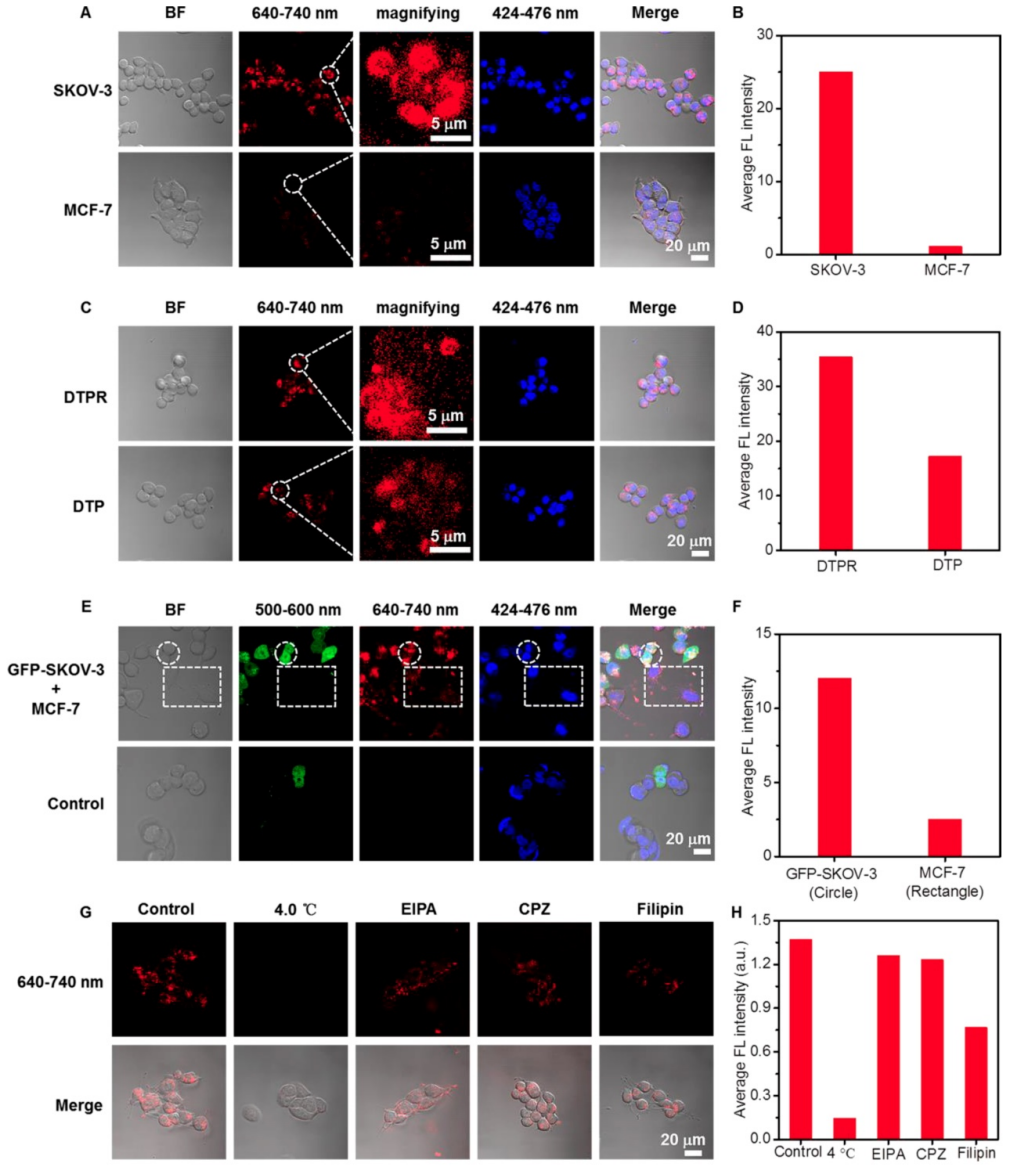
** (A)** CLSM images of SKOV-3 and MCF-7 cells after incubation with DTPR nanoparticles (10 μg/mL) for 4 h, respectively. **(B)** The corresponding average red fluorescence intensity of SKOV-3 and MCF-7 cells. **(C)** CLSM images of SKOV-3 cells after incubation with 10 μg/mL DTPR and DTP for 4 h, respectively. **(D)** The corresponding average red fluorescence intensity of SKOV-3 cells. **(E)** The CLSM images of co-cultured GFP-SKOV-3 and MCF-7 cells after incubation with 10 μg/mL DTPR for 4 h, and then 10 μM Hoechst 33258 for 30 min. **(F)** The average red fluorescence intensity of GFP-SKOV-3 (circle) and MCF-7 (rectangle) co-cultured cells incubating with DTPR. **(G)** CLSM and **(H)** corresponding average FL intensities of SKOV-3 cells incubated with 10 μg/mL DTPR before (control) and after treatment with low temperature (4.0 °C) or various inhibitors (CPZ, EIPA or filipin). DTPR channel: excitation wavelength, 808 nm; emission collected: 640-740 nm. GFP channel: excitation wavelength, 488 nm; emission collected: 500-600 nm. Hoechst 33258 channel: excitation wavelength, 720 nm; emission collected: 424-476 nm. Scale bars: 20 μm.

In the following, αvβ3 integrin low-expression MCF-7 cells were selected as the negative control 72, 73. As shown in **Figure 3A, 3B**, **Figure S24A, S24B, S25A, S25B**, DTPR exhibited much higher cellular internalization towards SKOV-3 cells than MCF-7 cells. Obvious fluorescence difference in cytoplasm between two cell lines clearly manifested that DTPR was highly selective for SKOV-3 cells. For comparison, the fluorescence intensity of DTPR was nearly 2-fold higher than that of DTP under the identical experimental conditions (**Figure 3C, 3D, Figure S24C, S24D, S25C, S25D**). Moreover, the targeting ability of DTPR was further proved by co-culturing experiment of MCF-7 and GFP-SKOV-3 cells (**Figure 3E, 3F, Figure S24E, S24F, S25E, S25F**). The red fluorescence was stronger in GFP-SKOV-3 cells (circle parts) than that in MCF-7 cells (rectangle parts). All the above results demonstrated that DTPR exhibited better selective uptake towards αvβ3 integrin over-expression SKOV-3 cells than αvβ3 integrin low-expression MCF-7 cells.

To clarify the cellular uptake mechanisms, we investigated the cellular internalization efficiency of DTPR nanoparticles in SKOV-3 cells by pretreating with low temperature and various inhibitors: filipin (inhibitor of caveolae-mediated endocytosis), 5-(N-ethyl-N-isopropyl)-amiloride (EIPA, inhibitor of macropinocytosis) and chlorpromazine hydrochloride (CPZ, inhibitor of clathrin-mediated endocytosis) 74-78. CLSM imaging results (**Figure 3G, 3H, Figure S26, S27, S28**) displayed that 4.0 °C treatment could seriously restrain the cell uptake of the nanoparticles, intimating their energy-dependent endocytosis (since 4.0 °C would lead to ATP deficiency). Furthermore, the fluorescence intensity of EIPA and CPZ treated cells did not decrease significantly compared with the control groups, indicating that cellular uptake was not influenced by either EIPA or CPZ. Nonetheless, the fluorescence intensity of filipin treatment reduced markedly, confirming that the internalization of DTPR nanoparticles was partly caveolae-mediated.

#### Photocytotoxicity

**Figure 4:**
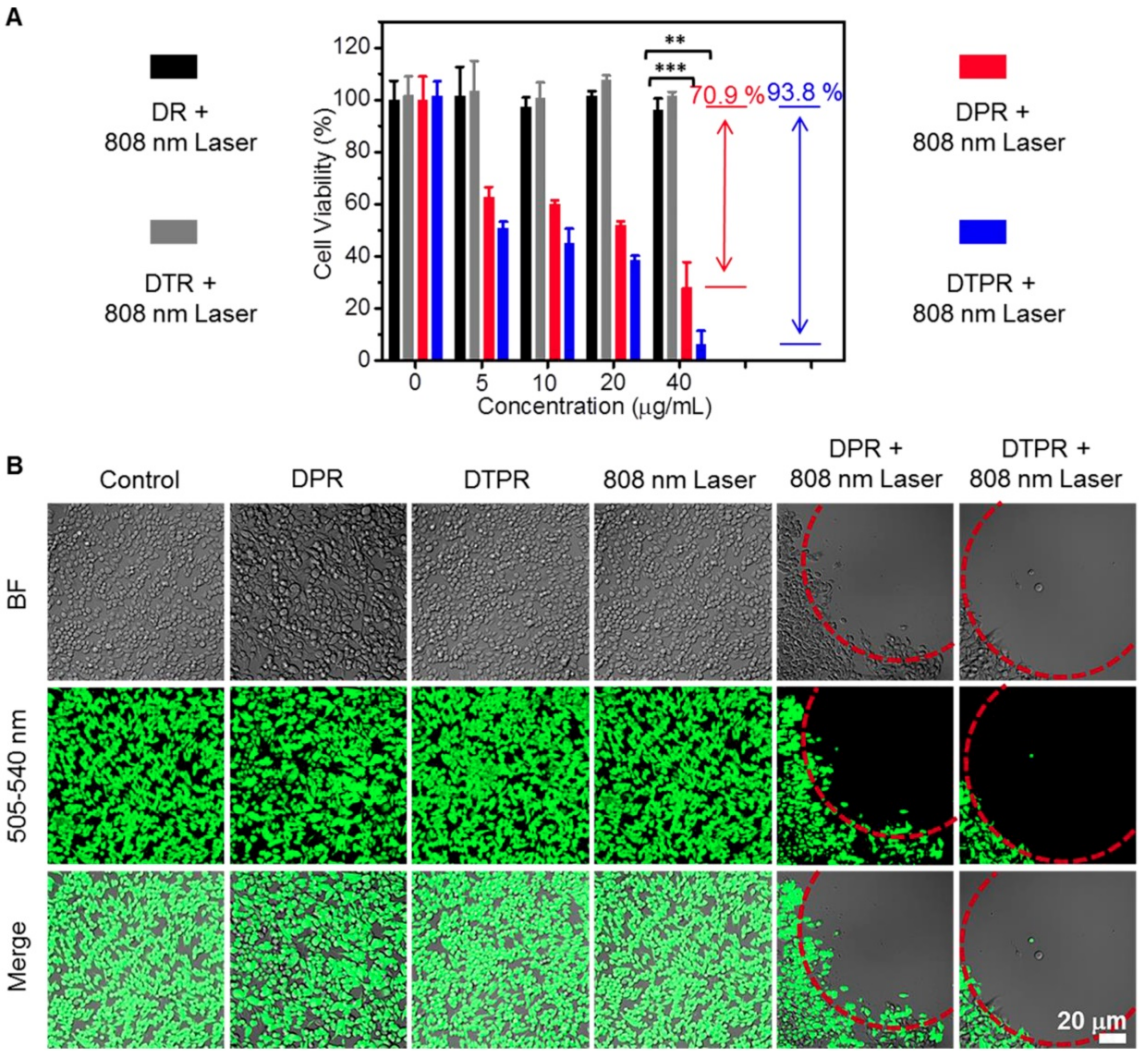
** (A)** Cell viabilities of SKOV-3 cells after DR, DTR, DPR, DTPR (0, 5, 10, 20 and 40 μg/mL)-induced photothermal ablation under 808 nm laser irradiation at power density of 1.1 W/cm^2^. All the data were presented as the average ± standard error (n = 5). **(B)** CLSM images of DPR, DTPR treated live cells after 808 nm laser irradiation. Green channel: excitation wavelength, 488 nm; emission collected: 505-540 nm, Scale bars: 20 μm. Statistical significance: ^**^P<0.01, ^***^P<0.001.

We next examined the cytotoxicity of DR, DTR, DPR and DTPR on SKOV-3 cells via MTT assay. In the absence of photo-irradiation, DR, DTR, DPR and DTPR did not induce observable cytotoxicity (**Figure S29**), indicating the excellent cytocompatibility. However, DTPR exhibited an irradiation concentration-dependent cytotoxicity against SKOV-3 cells, and possessed much stronger photocytotoxicity (93.8%) than DPR (70.9%) under the same condition (**Figure 4A**). Such results were ascribed to the fact that the FRET effect of DTPR obtained the additional heat and triggered much more severe photothermal injury to SKOV-3 cells. Subsequently, the PTT efficacy of DTPR was also analyzed by CMFDA staining assay which was used to label living cells. As shown in **Figure 4B**, nearly all of the SKOV-3 cells in the red circle were killed by the photothermal ablation after treated with DTPR under 808 nm laser irradiation. In contrast, control experiments (without any treatment, DPR only, DTPR only and 808 nm laser irradiation only) were not significantly affected. As for DPR, the cell survival rate was much higher than DTPR-treated group, further indicating that dual heat had much higher killing capacity than single heat. Not surprisingly, DR, DTR with various treatments exhibited negligible effects on the cell viability (**Figure S30**). Taken together, these results signified that the dual heat of DTPR triggered the most severe photocytotoxicity effect on cellular treatment, and was expected to play a vital role in tumor therapy.

### *In vivo* Studies of DTPR Nanoparticles

It has been reported that nanoparticles could be used for intravenous injection, the hemolysis rate should be less than 5% 60. Hence, before intravenous injection of DTPR and other control nanoparticles, the hemolysis test was carried out via evaluating the UV absorbance of hemoglobin. As shown in **Figure S31**, the hemolysis ratios were all below 3% at the concentrations of 100 μg/mL, which indicated that DTPR and other control nanoparticles were suitable for intravenous administration.

**Figure 5:**
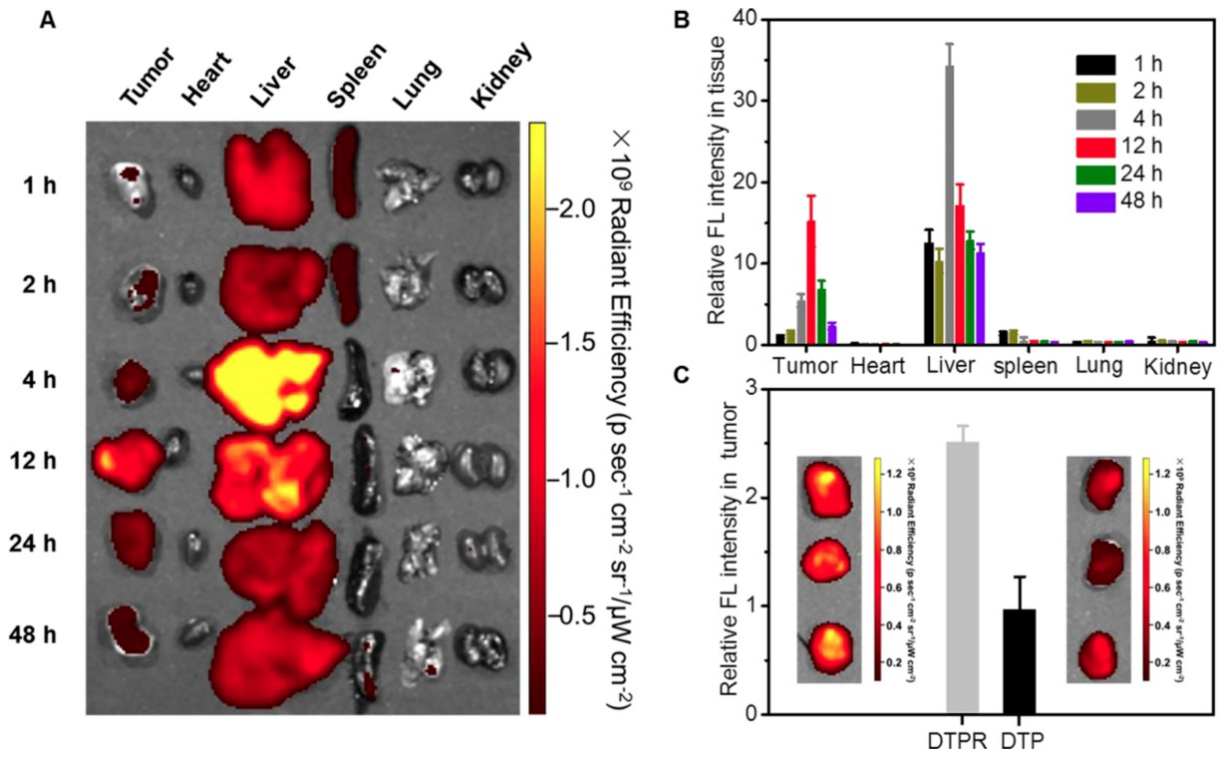
** (A)** *Ex vivo* images of dissected organs and tumors after intravenous injection with DTPR nanoparticles for varied time. **(B)** The corresponding accumulative amounts of different organs and tumors, which were the quantitative data of a). **(C)** The corresponding accumulative amounts of tumors after intravenous injection with DTP and DTPR nanoparticles for 12 h, respectively. Inset: *ex vivo* images of dissected tumors.

Next, the *in vivo* behavior of DTPR nanoparticles was explored using SKOV-3 tumor-bearing mouse model. After intravenous injection of DTPR nanoparticles, fluorescence images were obtained at various time points post-injection (1, 2, 4, 12, 24 and 48 h). From the results in **Figure 5A, 5B**, we could conclude that DTPR nanoparticles tended to accumulate in tumor sites over time and the maximum uptake of nanoparticles was at 12 h post-injection. Thus, 12 h post-injection was chosen as ideal injection time to perform the *in situ* therapy. Imprtantly, after injection for 12 h, the *ex vivo* fluorescent images of the excised tumors validated that DTPR accumulation at tumor sites was 2.6-fold higher than that of DTP nanoparticles (**Figure 5C**). These results indicated that DTPR nanoparticles could be remarkably enriched into tumor regions through the enhanced active targeting and provide a crucial prerequisite for superior PTT efficacy in the animal model.

### *In vivo* Anticancer Effect

**Figure 6:**
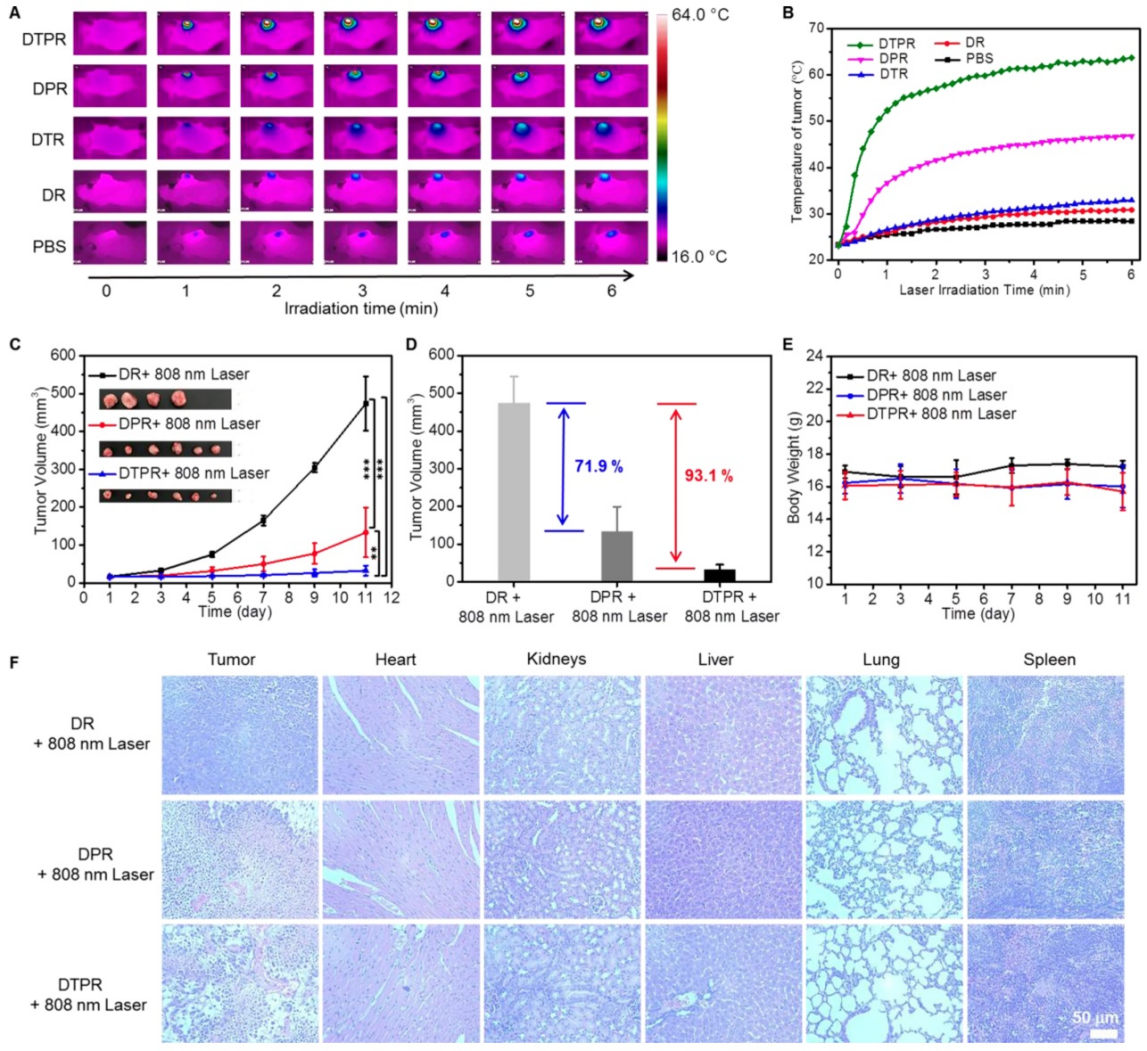
** (A)** Infrared thermal images of SKOV-3 tumor-bearing mice under 808 nm laser irradiation (0.8 W/cm^2^) for different times after intravenous injection of 200 μL nanoparticles (100 μg/mL) and PBS, respectively. **(B)** The corresponding temperature changes in the tumor sites correspondingly as the irradiating time, which was the quantitative data of **(A)**. **(C)** Time-dependent tumor growth curves after various treatments as indicated. Inset: photos of tumors after various treatments were taken at day 11. **(D)** After treated for 11 days, the volume of tumors decreased in different treatment groups compared with the control group. **(E)** Body weight changes of SKOV-3 tumor-bearing mice (“DR + 808 nm laser” groups, n = 4, “DPR/DTPR + 808 nm laser” groups, n = 6). **(F)** Histological changes of tumors, heart, kidney, liver, lung and spleen in different treatment groups were detected by H&E staining. Scale bars: 50 μm. Statistical significance: ^*^P<0.05, ^**^P<0.01, ^***^P<0.001.

To verify the heating capacity of our nanoparticles *in vivo*, the tumor temperatures of mice for each group were recorded after intravenous injection with PBS and nanoparticles. As shown in **Figure 6A, 6B**, negligible temperature changes were observed in the tumors of PBS, DR and DTR groups, while tumors in DPR nanoparticles group exhibited a temperature increase of about 49.0 °C. Notably, a much higher temperature (66.0 °C) was observed from the tumors in DTPR-treated mice, attributing to 808 nm laser-actived FRET-induced dual heat effect that provoked remarkable hyperthermia.

The tumor-bearing mice were randomly divided into nine groups and tumor volumes were monitored for 11 days to examine the *in vivo* anti-tumor efficacies of DTPR nanoparticles (**Table S2**). As depicted in **Figure 6C, Figure S32A, S32B**, the mice treated with “PBS only”, “PBS + 808 nm laser”, “DR only”, “DR + 808 nm laser”, “ DPR only”, “DTPR only” and “DTR + 808 nm laser” failed to inhibit tumor growth. Nevertheless, the “DPR + 808 nm laser” group treated via single PTT exhibited a favourable antitumor efficacy, with the average tumor volume decreased by 71.9% on day 11. The most prominent antitumor efficacy came from the “DTPR + 808 nm laser” group, whose mean tumor volume was severely reduced by 93.1% on day 11 (**Figure 6D**). Furthermore, after treatment, tumor volume in “DTPR + 808 nm laser” group was less than one third of that in “DPR + 808 nm laser” group. Such highly efficient tumor growth inhibition should be attributed to not only the 808 nm laser-activated dual PTT effect but also the impressive tumor uptake ability of RGD-targeted nanoparticles. Most importantly, all the mice exhibited negligible weight fluctuations, thus suggesting the low adverse effects of these treatments (**Figure 6E, Figure S32C**).

The therapeutic effect of each group was further evaluated at the microscopic level. The mice in all nine groups of this experiments were sacrificed. Tumors as well as other organs including kidney, heart, spleen, liver and lung were dissected and sliced for hematoxylin and eosin (H&E) staining 79-82. The results in **Figure 6F, Figure S32D** affirmed that the mice treated with “PBS only”, “PBS + 808 nm laser”, “DR only”, “DR + 808 nm laser”, “ DPR only”, “DTPR only” and “DTR + 808 nm laser” did not exhibit obvious tumor necrosis, while “DPR + 808 nm laser” treatments caused considerable damage to the tumor region but no significant damage to the normal organs. In particular, “DTPR + 808 nm laser” group was most effective in inducing apoptosis and inhibiting tumor cells proliferation.

## Conclusion

In conclusion, a novel “nanococktail” DTPR nanoparticles was successfully developed for 808 nm-activated image-guided dual PTT. In this system, AIEgens (T) was partially used as imaging probe as well as fluorescence resonance energy donor, while SPs (P) was served as photothermal agent and fluorescence resonance energy receptor. Such dual PTT was achieved by FRET effect from T to P that generated heat plusing its original PTT effect of P upon a single 808 nm laser excitation. In solution, upon 808 nm laser irradiation, DTPR nanoparticles were able to efficiently transfer light to heat with the conversion efficiency of 60.3%, which was 1.9-fold of that towards DPR (31.5%). *In vitro*, DTPR possessed targeting ability toward αvβ3 integrin over-expressed SKOV-3 cells rather than αvβ3 integrin low expression MCF-7 cells. DTPR with dual PTT displayed stronger cytotoxicity (93.8%) than DPR nanoparticles with single PTT (70.9%) under the same condition against SKOV-3 cells. Moreover, *In vivo* photothermal experiments confirmed that DTPR nanoparticles targeted the tumor and largely suppressed tumor growth under 808 nm laser irradiation while causing little damage to the normal tissues of mice. In summary, *in vitro* and *in vivo* anti-tumor experiments have proved that the antitumor effect of dual PTT was better than that of single PTT and dual PTT was a simple and effective strategy for cancer treatment.

## Supplementary Material

Supplementary materials and methods, scheme, figures, and tables.
