# Supplementary material for: Nanococktail Based on AIEgens and Semiconducting Polymers: A Single Laser Excited Image-Guided Dual Photothermal Therapy
Source: Theranostics. 2020 Jan 12;10(5):2260–72. doi: 10.7150/thno.41317 (PMC7019155; doi:10.7150/thno.41317)
Supplement: Supplementary file 1 — Supplementary materials and methods, scheme, figures, and tables. [file thnov10p2260s1.pdf]

Supporting Information

Nanococktail Based on AIEgens and Semiconducting Polymers: A Single Laser Excited Image-Guided Dual Photothermal Therapy

Zi Long*[1], Jun Dai*[2], Qinyu Hu [1], Quan Wang [1], Shijie Zhen[4], Zujin Zhao[4], Zitong Liu[3], Jing-Jing Hu,[1] Xiaoding Lou[1]✉, Fan Xia [1]

1. Engineering Research Center of Nano-Geomaterials of Ministry of Education, Faculty of Materials Science and Chemistry, China University of Geosciences, Wuhan 430074, China.
2. Department of Obstetrics and Gynecology, Tongji Hospital, Tongji Medical College, Huazhong University of Science and Technology, Wuhan 430074, China.
3. Beijing National Laboratory for Molecular Sciences, Organic Solids Laboratory, Institute of Chemistry, Chinese Academy of Sciences, Beijing 100190, China.
4. State Key Laboratory of Luminescent Materials and Devices, Center for Aggregation-Induced Emission, South China University of Technology, Guangzhou 510640, China.

* These authors contributed equally to this work.

✉ Corresponding author: E-mail address: louxiaoding@cug.edu.cn (X. Lou)

Table of Contents

1. Materials and Methods

1.1. General Information.

1.2. Detection of ROS in Solution.

1.3. Photothermal Performance of Nanoparticles.

1.4. Cell Culture.

1.5. Cellular Imaging.

1.6. Endocytosis Mechanism.

1.7. Cytotoxicity Studies.

1.8. PTT-mediated Apoptosis Assay.

1.9. Hemolysis Test.

1.10. *In vivo* Biodistribution of DTPR.

1.11. Photothermal Imaging.

1.12. Therapeutic Effect *in vivo*.

2. Supporting Scheme, Figures and Tables

1. Materials and Methods

1.1. General Information

9,10-anthracenediylbis(methylene)dimalonic acid (ABDA) was supplied by Sigma-Aldrich. DSPE-PEG$_{2000}$-Maleimide was provided by Xi'an ruixi Biological Technology Co., Ltd. (Xi'an, China). 5-Chloromethylfluorescein diacetate (CMFDA) was supplied by Yeasen Biotech Co., Ltd. (Shanghai, China). Filipin was bought from Santa Cruz biotechnology, while chlorpromazine hydrochloride (CPZ) and ethylisopropylamiloride (EIPA) were obtained from MedChemExpress (USA). The peptide RGD (RGDFGGRRRRC) was synthesized by GL Biochem Ltd. (Shanghai, China). All other chemicals and reagents were obtained from commercial sources and used as received without further purification. Deionized water (18.2 MΩ·cm) used in all experiments was obtained from a Milli-Q water-purification system (Millipore Corp., Billerica, MA, USA). The SKOV-3, MCF-7 cells were purchased from Boshide (Wuhan, China). Culture medium (DMEM), fetal bovine serum (FBS) and phosphate buffer saline (PBS, pH 7.4) were all provided by HyClone Thermofisher (Beijing, China).

Polymer molecular weight was measured with PL-GPC 220 high temperature chromatograph equipped with an IR5 detector at 140 ℃; polystyrene was utilized as the calibration standard and 1,2,4-trichlorobenzene as eluent (1 mL/min). UV-Vis absorption spectra were taken on an Agilent Cary 60 UV/Visible Spectrometer. All the fluorescence measurements were performed on a FS5 spectrofluorometer (Edinburgh Instruments), except the two-photon Fluorescence spectra were recorded on a Horiba Fluoromax-4 fluorescence spectrophotometer. The dynamic light scattering (DLS) size distribution and zeta potential of particles were recorded by a Malvern Instruments Zetasizer Nano ZS90. Field emission high-resolution scanning electron microscope (FESEM) were recorded by Hitachi SU-8010. Transmission electron microscope (TEM) images were obtained with a FEI Tecnai G2 12 TEM instrument with an accelerating voltage of 100 kV. MTT assay was obtained on an

Infinite M200 PRO Microplate Reader (Tecan Austria). Confocal laser scanning microscopy images were recorded using LSM 880 confocal microscopy (Carl Zeiss), equipped with a FemtoSecond Laser (Coherent Inc.). A 20 × oil or 63 × oil objective was used in the imaging process.

1.2. Detection of ROS in Solution

The ROS generation was studied by using ABDA as an indicator as the absorbance of ABDA decreases upon reaction with ROS. 10 μL of ABDA solution (4.5 mg/mL in DMSO) was added to different nanoparticles, then 1 mL mixed solution in Petri dish was irradiated with 808 nm laser (1.1 W/cm^2). The decomposition of ABDA was monitored by the absorbance decrease. The absorbance of ABDA at 400 nm was recorded for different durations of 808 nm laser irradiation to obtain the decay rate of the photosensitizing process.

1.3. Photothermal Performance of Nanoparticles

Photothermal performance of nanoparticles was measured and analyzed by irradiating a 1.5 mL EP tube containing 100 μL different nanoparticles dispersion with different concentrations. NIR laser was produced using an 808 nm power multimode pump laser (Changchun New Industries Optoelectronics Tech Cc., Ltd, china). The temperature and thermal images of the irradiated aqueous dispersion and cells were recorded on an infrared thermal imaging instrument (FLIR ™ E85 camera, Japan).

1.4. Cell Culture

SKOV-3, MCF-7 cells were cultured in DMEM medium in an atmosphere of 5% CO_2 at 37 ℃. The medium contained 1% antibiotics (penicillin-streptomycin, 10,0000 U/mL) and 10% heat-inactivated FBS. Before experiments, the cells were pre-cultured until confluence was reached.

1.5. Cellular Imaging

For confocal laser scanning microscopy (CLSM) imaging, cells were seeded into cell culture dishes at a density of 2×10^4 in growth medium (DMEM supplemented with 10% FBS).

After an overnight incubation, the cells were washed with phosphate buffer saline (PBS, pH 7.2-7.4) for three times. A solution of the indicated nanoparticles in medium was then added, and the cells were incubated in a 5% CO_2 atmosphere at 37 ℃ for further usage. For co-culture cells CLSM, the same number (2×10^4 of each dish) of GFP-SKOV-3 cells and MCF-7 cells were co-culture in the medium of DMEM medium at 37 ℃ in a humidified atmosphere containing 5% CO_2.

1.6. Endocytosis Mechanism

To study the endocytosis mechanism of DTPR nanoparticles, SKOV-3 cells were incubated with DTPR at 4 ℃ to study the effect of low temperature on the cellular endocytosis of the nanoagents. Besides, we investigated the possible endocytosis pathways of DTPR nanoparticles using the following inhibitors: filipin (1 μg/ml), chlorpromazine (10 μg/ml), EIPA (10 μg/ml). After separately pre-incubating of these inhibitors for 30 min in serum-free medium, cells were then treated with DTPR nanoparticles (10 μg/mL) for 4 h, and then analyzed by CLSM imaging.

1.7. Cytotoxicity Studies

The metabolic activities of SKOV-3 cells were evaluated using MTT assays. SKOV-3 cells were seeded in 96-well plates at an intensity of 5×10^3 cells/mL, respectively. After 80% confluence, the old medium was replaced by different nanoparticles in DMEM suspension at various concentrations. After 24 h incubation, the suspensions were replaced by fresh DMEM containing 10% FBS and 1% penicillin-streptomycin. The selected wells were exposed to 808 nm laser irradiation (1.1 W/cm^2, 3 min). The cells were further cultured for 24 h, and then washed with 1× PBS buffer and 20 μL of freshly prepared MTT (5 mg/mL) solution was added into each well. The MTT medium solution was carefully removed after 3 h incubation in the incubator. DMSO (150 μL) was then added into each well and the plate was gently shaken for 10 min at room temperature to dissolve all the precipitates formed. The absorbance

of MTT at 570 nm was monitored by the microplate reader. Cell viability was expressed by the ratio of the absorbance of the cells incubated with nanoparticles to that of the cells incubated with culture medium only.

1.8. PTT-mediated Apoptosis Assay

SKOV-3 cells were incubated with nanoparticles (40 μg/mL) for 4 h. After washing three times using PBS, the cells were treated with 808 nm laser irradiation (1.1 W/cm^2, 5 min), the area of illumination was controlled by masking. After removal of the medium, the adherent cells were rinsed with PBS for three times. Then SKOV-3 cells were stained with CMFDA for 15 min and washed three times with fresh culture medium to remove dead cells. Fluorescent images were visualized under fluorescent microscope.

1.9. Hemolysis Test

The release of hemoglobin from BALB/c nude mice blood cells was used to evaluate the hemolytic activities of DR, DTR, DPR, and DTPR nanoparticles by spectrophotometry. The blood samples were centrifugated and resuspended in normal saline to get the red blood cells (RBCs 2%). 100 μL RBCs suspension mixed with 100 μL ultrapure water and 100 μL normal saline solution were regarded as positive control (producing 100% hemolysis) and negative control (producing no hemolysis), respectively. Whereafter, 100 μL of different nanoparticles solutions were added into the mixture of 100 μL RBCs suspension, respectively. After kept at 37 ℃ for 3 h, all the samples were centrifuged. The absorbance of supernatants was measured with UV spectrophotometer and the normal saline was used as blank. The hemolysis ratio of RBSs was calculated using the following formula: hemolysis (%) = (A_{sample} -$A_{negative}$) / ($A_{positive}$ -$A_{negative}$) × 100%, where A_{sample}, $A_{negative}$, and $A_{positive}$ refered to the absorption of nanoparticles sample solution, negative control, and positive control at 570 nm, respectively.

1.10. *In vivo* Biodistribution of DTPR

BALB/c nude mice (4-6 weeks old) were purchased from Beijing Vital River Laboratory

Animal Technology Co., Ltd. The mice were housed under specific-pathogen-free conditions within the animal care facility at Experimental Animal Center, Tongji Medical College, Huazhong University of Science and Technology, and the animal experiments were performed in compliance with the Guidance Suggestions for the Care and Use of Laboratory Animals that approved by the ethics committee of Huazhong University of Science and Technology. A cell suspension of 1×10^8 cells/mL was prepared. The tumor-bearing mice were obtained by subcutaneous injection of cell suspension (2×10^7 cells). When tumors grew up to appropriate volume (tumor volume = width^2 × length/2), the mice were tail vein injected with 200 μL of DTPR (100 μg/mL). At predetermined time points (1, 2, 4, 12, 24, and 48 h post-injection), the tumors and main organs (heart, liver, spleen, lung and kidney) were monitored by the IVIS Spectrum (PerkinElmer) (λ_{ex} =535 nm, λ_{em} =680 nm). To examine the targeting of RGD peptides, SKOV-3 tumor-bearing mice were tail vein injected with 200 μL, 100 μg/mL DTP and DTPR, respectively. Post-injection 12 h, tumors were also monitored by the IVIS Spectrum (λ_{ex} =535 nm, λ_{em} =680 nm).

1.11. Photothermal Imaging

SKOV-3 tumor-bearing mice were tail vein injected with 200 μL PBS, DR, DTR, DPR and DTPR (100 μg/mL), respectively. Post-injection 12 h, tumors of the mice were irradiated for 6 min with 808 nm laser (0.8 W/cm^2). The temperature changes were recorded with a Thermal imaging equipment.

1.12. Therapeutic Effect *In vivo*

BALB/c nude mice bearing SKOV-3 tumors were invoked as model. When tumors grew up to about 15-20 mm^3 in volume, the mice were randomly assigned in nine groups: (a) PBS only group, (b) PBS+808 nm laser group, (c) DR only group, (d) DR+808 nm laser group, (e) DTR +808 nm laser group, (f) DPR only group, (g) DPR +808 nm laser group, (h) DTPR only group, (i) DTPR +808 nm laser group. Then 200 μL nanoparticles were tail vein injected once

per three days. For PTT groups, tumors were exposed to 808 nm laser at 0.8 W/cm^2 for 6 min. After treatments, body weight and tumor sizes of each mouse were monitored by a digital caliper at a unique time point every day. To further explore the effect of therapy, 11 days after various treatments, the mice were sacrificed, and the tumors and other major organs (heart, liver, spleen, lung, and kidney) were collected for further analysis. The tissue sections were stained with hematoxylin-eosin (H&E) following the standard protocol and then pathologically examined under a microscope.

2. Supporting Scheme, Figures and Tables

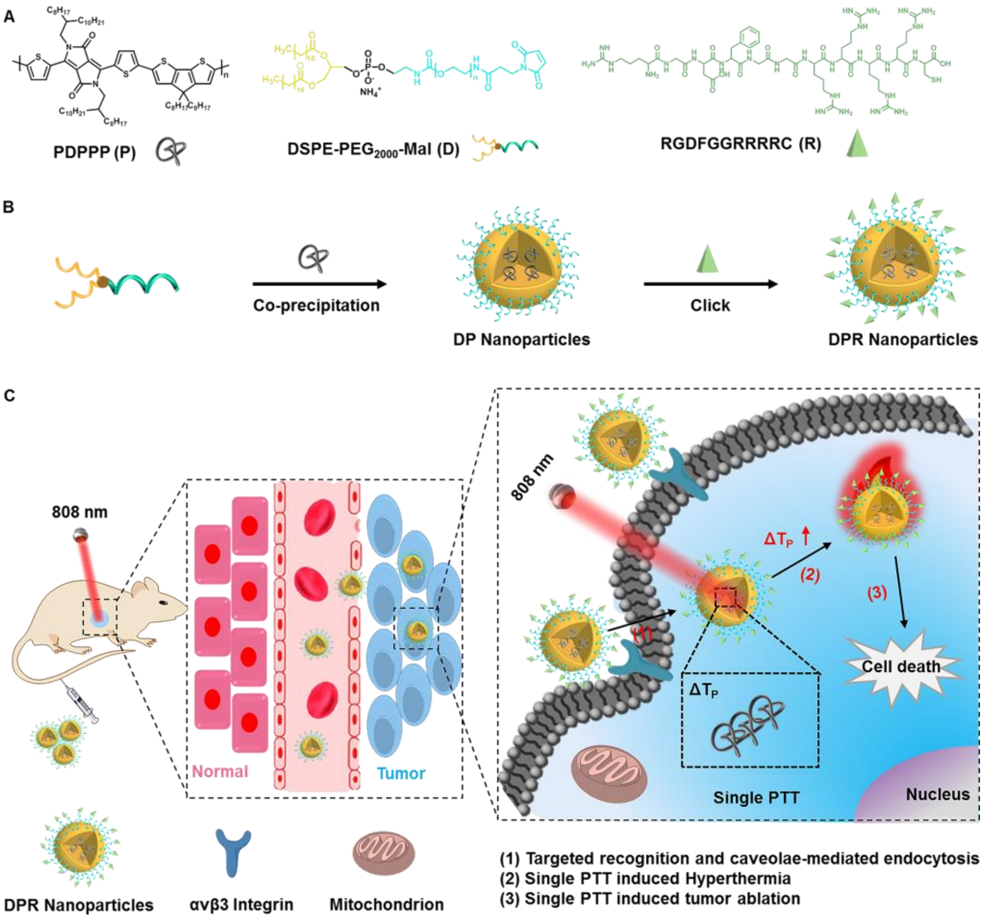

Scheme S1. Schematic showing the general design of "nanococktail" DPR nanoparticles for 808 nm-activated single PTT. **(A)** Chemical structures of P, D, R. **(B)** Schematic illustration of DPR nanoparticles preparation. **(C)** DPR nanoparticles were applied to single PTT via 808 nm-activated thermal effect.

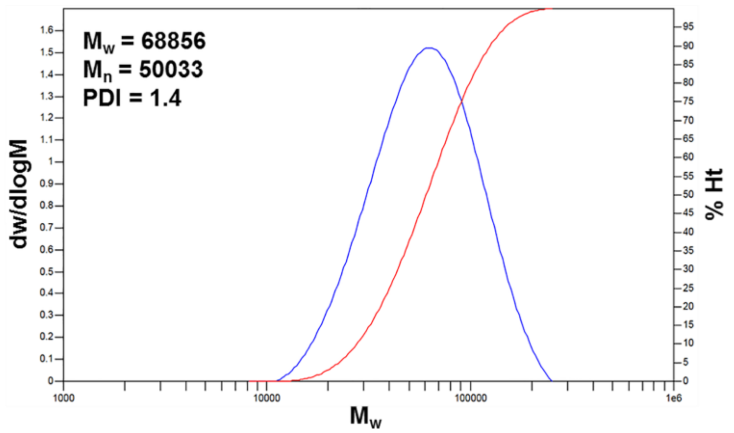

Figure S1. Gel Permeation Chromatography (GPC) traces of P.

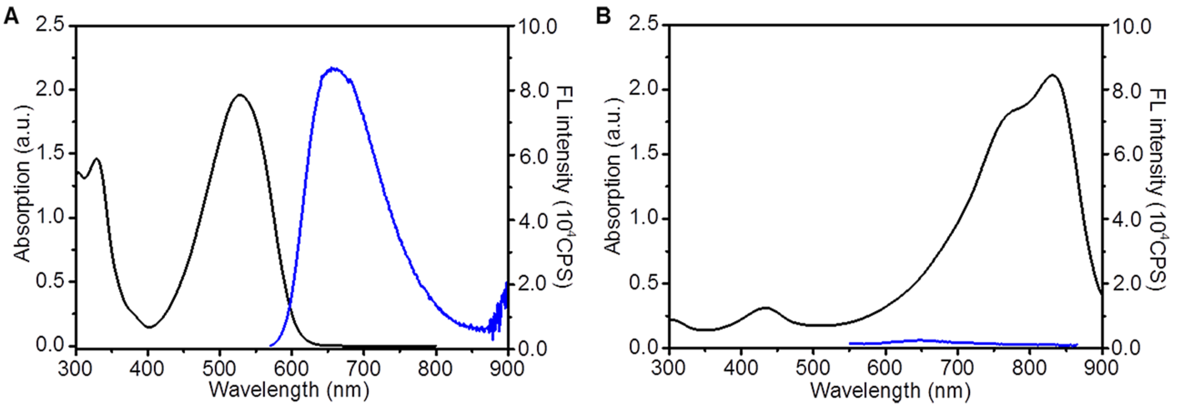

Figure S2. (A) Absorption (black) and FL spectra (blue) of T in 1% THF. **(B)** Absorption (black) and FL (blue) spectra of P in 1% THF, λ_{ex} = 530 nm.

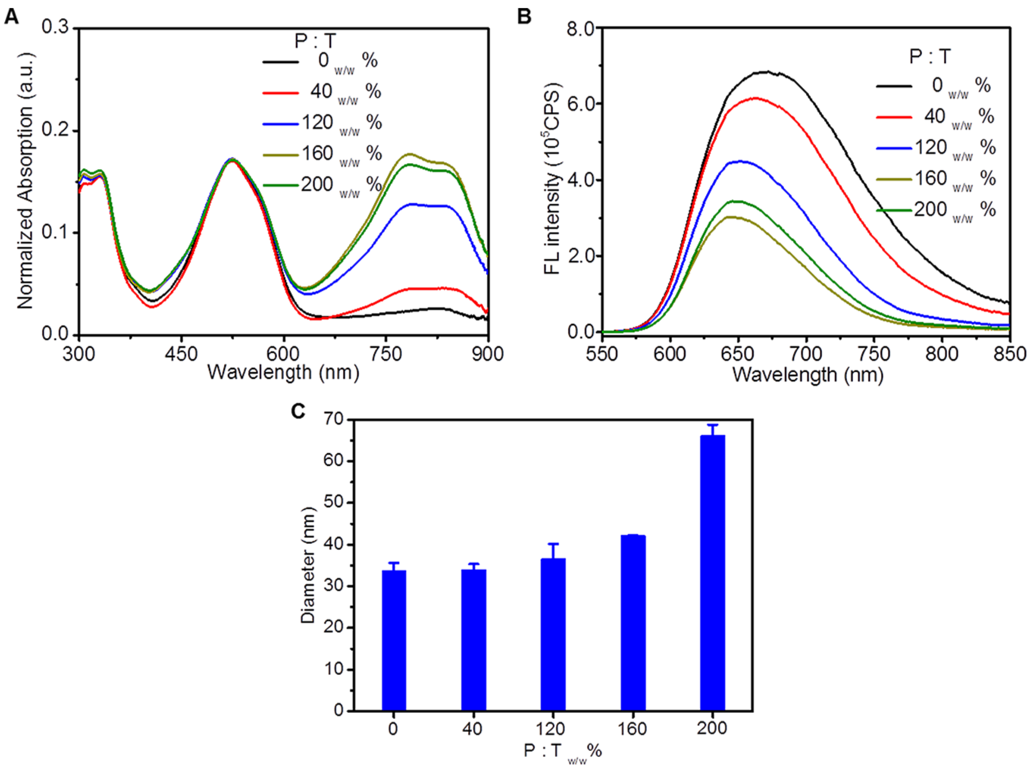

Figure S3. (A) Absorption spectra of DTP with different doping amount. **(B)** FL of DTP with different doping amount, λ_{ex} = 530 nm. **(C)** The DLS sizes of DTP nanoparticles with different doping amount. (The concentration of T was 0.5 mg/mL, while P was from 0.2 mg/mL to 1 mg/mL).

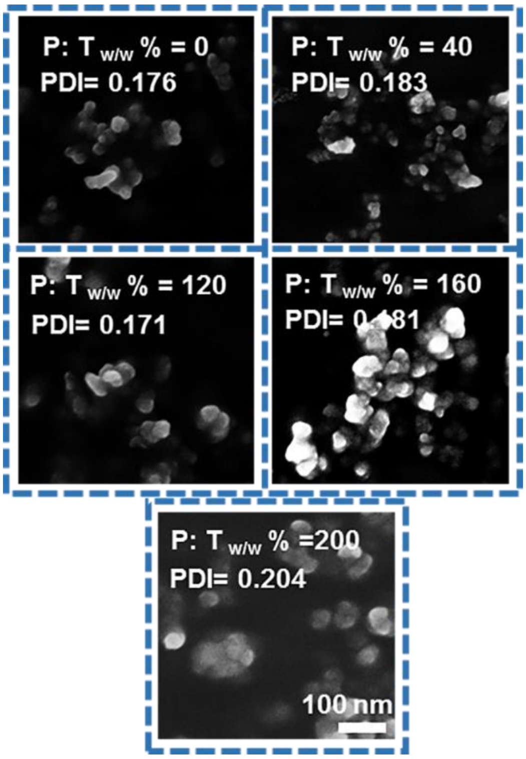

Figure S4. SEM images of DTP nanoparticles with different doping amount. Scale bar: 100 nm.

Samples	Encapsulation matrix	Encapsulation components		Targeting peptide
	D	T	P	R (RGDFGGRRRRC)
D	+	-	-	-
DT	+	+	-	-
DP	+	-	+	-
DTP	+	+	+	-
DR	+	-	-	+
DTR	+	+	-	+
DPR	+	-	+	+
DTPR	+	+	+	+

Table S1. D encapsulated different components with R or without R modification.

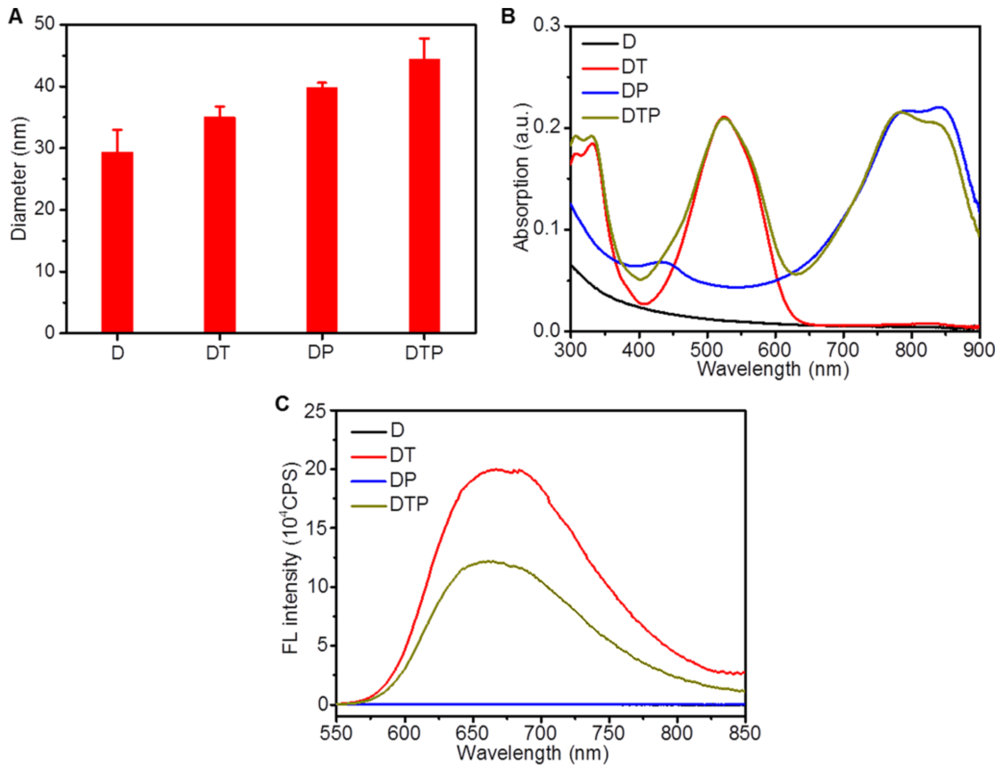

Figure S5. **(A)** DLS sizes, **(B)** absorption spectra and **(C)** FL spectra of D, DT, DP and DTP nanoparticles in aqueous solution. λ_{ex} = 530 nm.

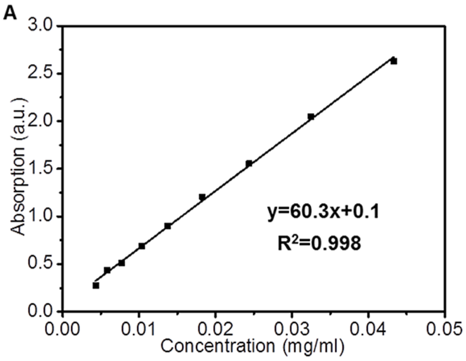

Equation	y=a+b*x		
Weight	No Weighting		
Pearson's r	0.999		
Adj.R-Square	0.998		
		Value	Standard Error
	Intercept	0.1	0.02
	Slope	60.3	0.9

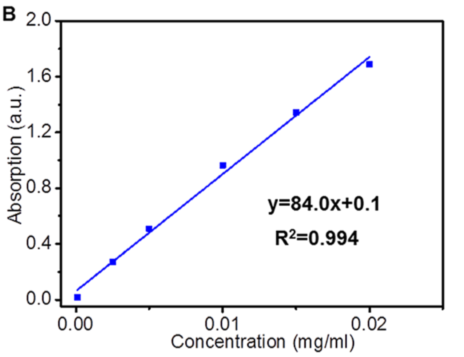

Equation	y=a+b*x		
Weight	No Weighting		
Pearson's r	0.997		
Adj.R-Square	0.994		
		Value	Standard Error
	Intercept	0.1	0.03
	Slope	84.0	3.0

Figure S6. (A) The standard curve of T, the table on the right was the relevant parameters of the curve. T was added 0.5 mg, the actual load of T was calculated to be 0.33 mg. **(B)** The standard curve of P, the table on the right was the relevant parameters of the curve. P was added 0.8 mg, the actual load of P was calculated to be 0.6 mg.

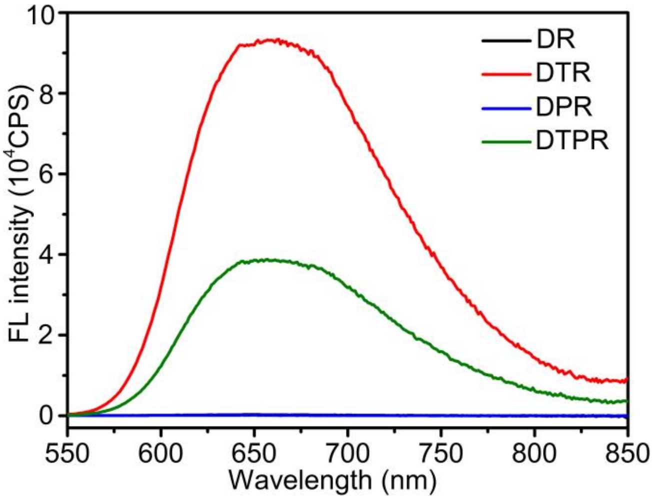

Figure S7. FL spectra of DR，DTR，DPR, DTPR nanoparticles in aqueous solution, λ_{ex} = 530 nm.

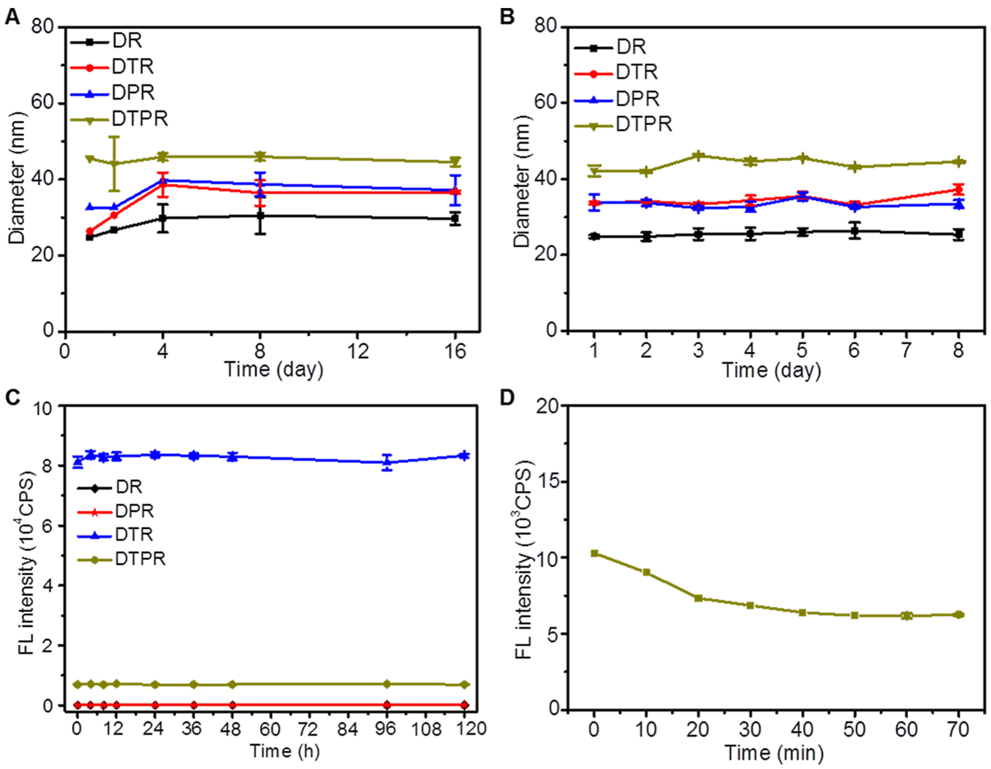

Figure S8. (A) Stability study of DR, DTR, DPR and DTPR nanoparticles in aqueous solution after storage under dark over a period of 16 days. **(B)** Stability study of DR, DTR, DPR and DTPR nanoparticles in 10% FBS containing solution. **(C)** The fluorescence stability of DR, DPR, DTR and DTPR nanoparticles in aqueous solution after 120 h, λ_{ex} = 530 nm. **(D)** The photostability of DTPR upon 808 nm laser irradiation for different time, λ_{ex} = 530 nm.

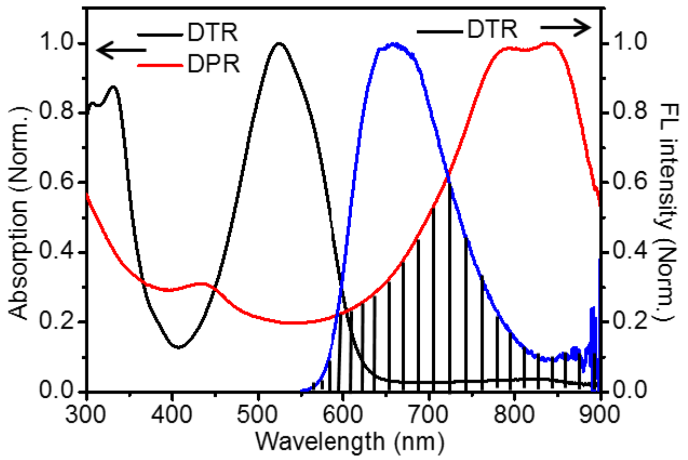

Figure S9. Normalized absorption and FL spectra of DTR and DPR nanoparticles. λ_{ex} = 530 nm.

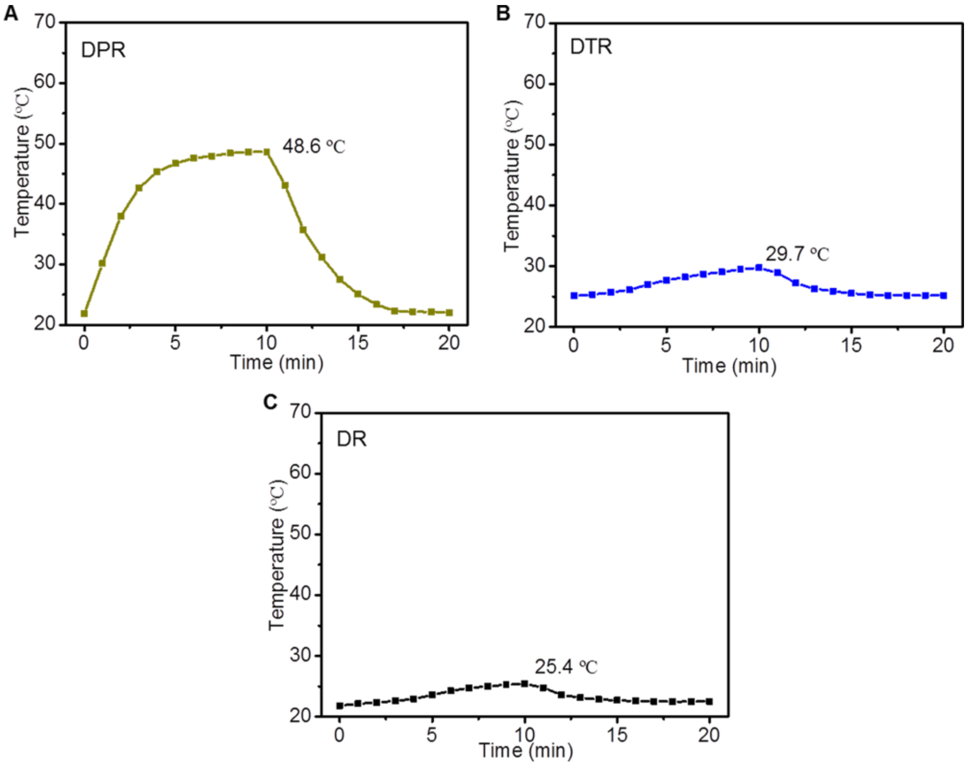

Figure S10. Photothermal effect of 10 μg/mL aqueous dispersion of **(A)** DPR, **(B)** DTR, **(C)** DR under irradiation with an 808 nm laser for 10 min (1.1 W/cm^2).

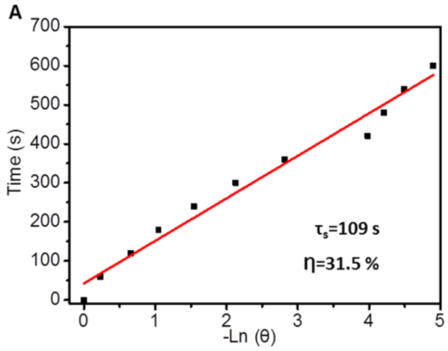

Equation	y=a+b*x		
Weight	No Weighting		
Pearson's r	0.990		
Adj.R-Square	0.977		
		Value	Standard Error
	Intercept	42.5	15.5
	Slope	109	5.3

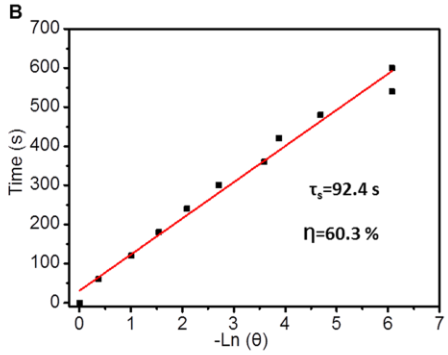

Equation	y=a+b*x		
Weight	No Weighting		
Pearson's r	0.993		
Adj.R-Square	0.984		
		Value	Standard Error
	Intercept	31.1	13.2
	Slope	92.4	3.7

Figure S11. Time constant (τ_s) of **(A)** DPR, **(B)** DTPR for the heat transfer from the system determined by applying the linear time data from the cooling period. The table on the right was the relevant parameters of the curve **(A)** and **(B)**.

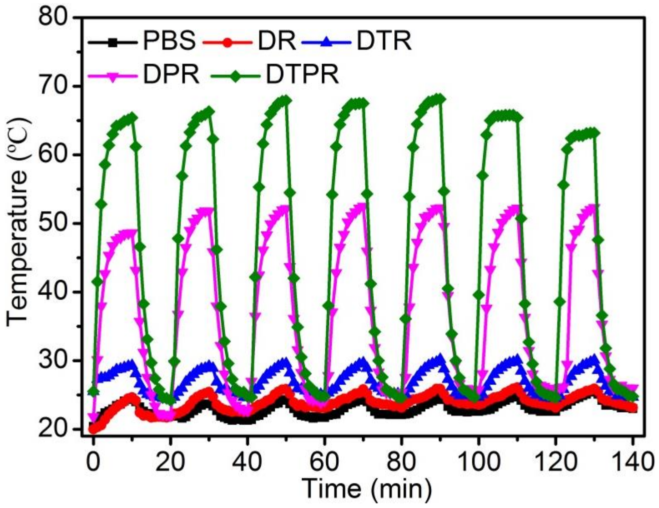

Figure S12. Heating curves of PBS, 10 μg/mL DR, DTR, DPR and DTPR suspension for seven on/off cycles (1.1 W/cm^2) under the irradiation with an 808 nm laser.

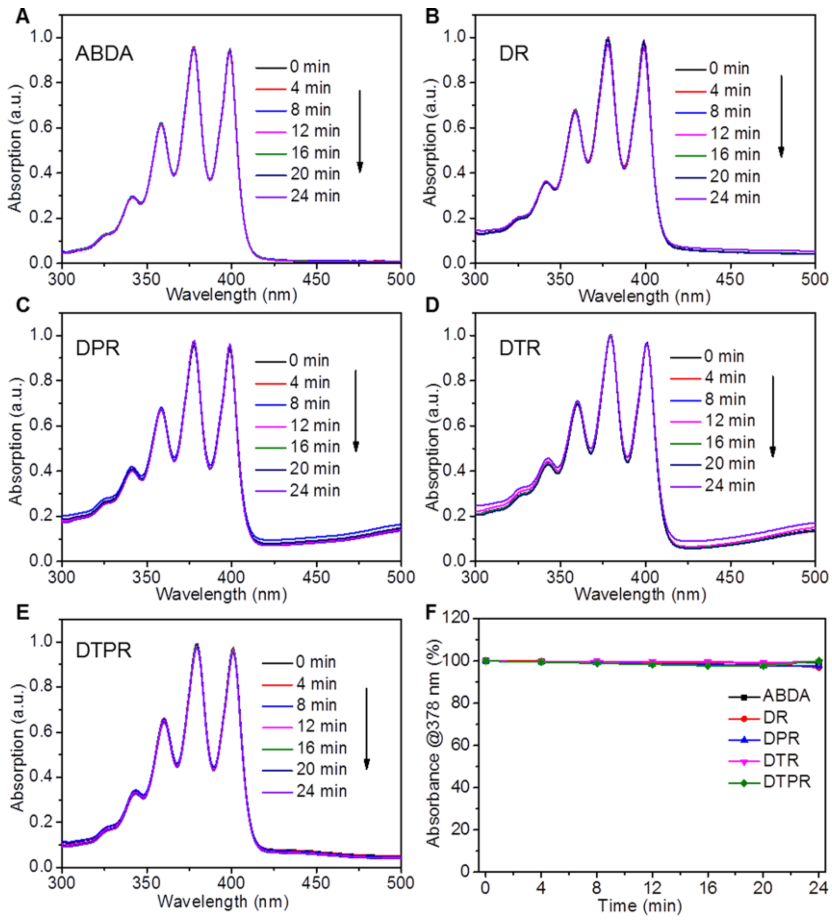

Figure S13. Absorption spectra of ABDA after light-decomposition by ROS generated from **(A)** ABDA, **(B)** DR, **(C)** DPR, **(D)** DTR, **(E)** DTPR upon 808 nm laser irradiation (1.1 W/cm^2). **(F)** Normalized degradation rates of ABDA in the presence of DR, DPR, DTR, DTPR.

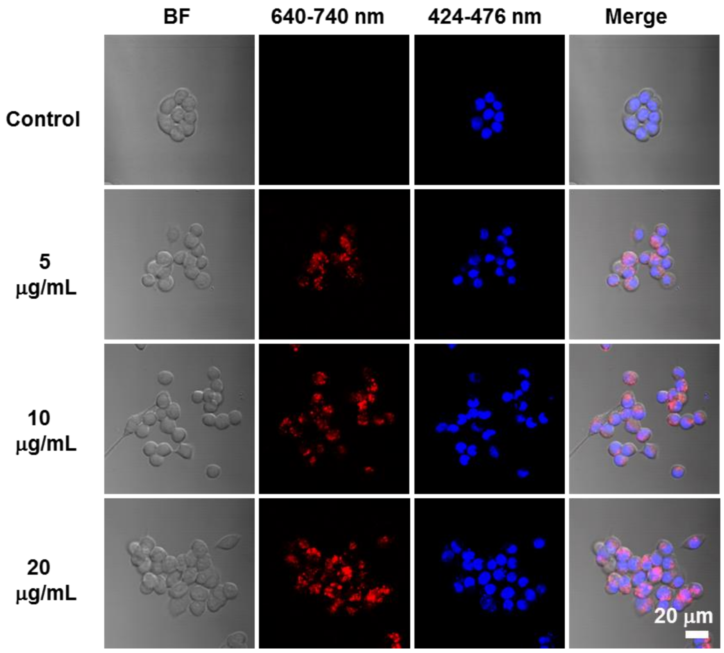

Figure S14. CLSM images of SKOV-3 cells with different concentrations of 5 μg/mL, 10 μg/mL, 20 μg/mL DTPR incubated for 4 h in cell culturing condition and the nuclei were stained by Hoechst 33258. Red channel: excitation wavelength, 808 nm; emission collected: 640-740 nm. Blue channel: excitation wavelength, 720 nm; emission collected: 424-476 nm. Scale bar: 20 μm.

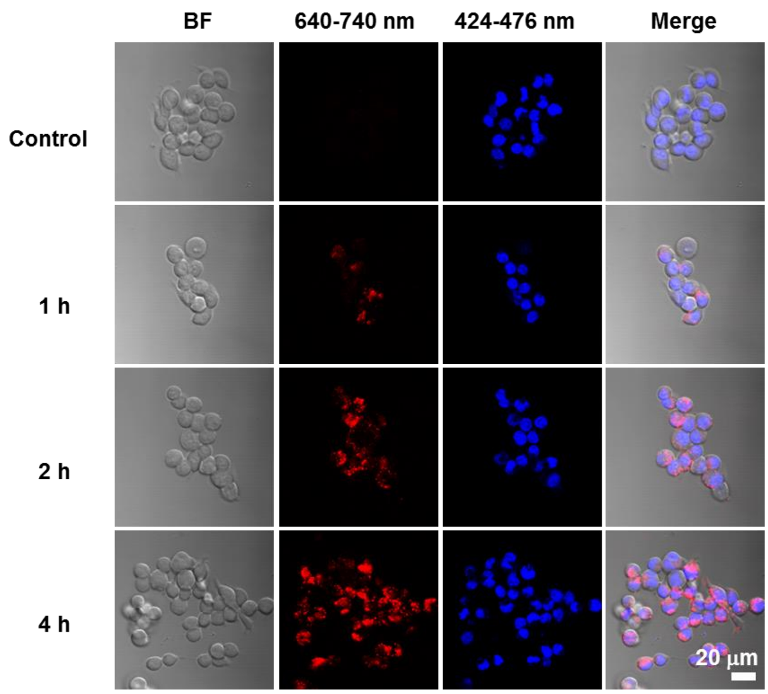

Figure S15. CLSM images of SKOV-3 cells incubated with 10 μg/mL DTPR for 1 h, 2 h, 4 h in cell culturing condition and the nuclei were stained by Hoechst 33258. Red channel: excitation wavelength, 808 nm; emission collected: 640-740 nm. Blue channel: excitation wavelength, 720 nm; emission collected: 424-476 nm. Scale bar: 20 μm.

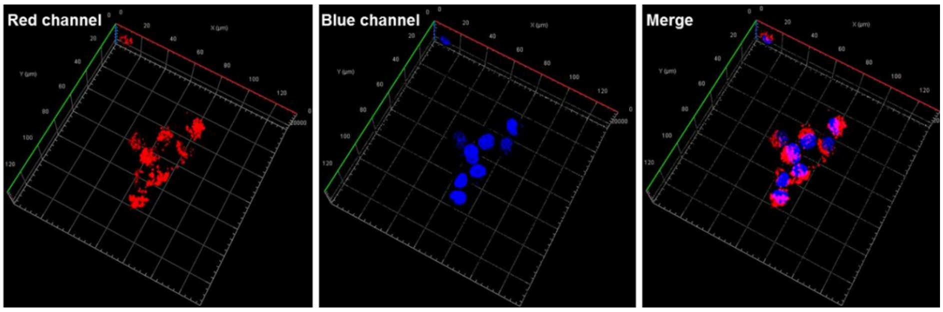

Figure S16. The three-dimensional CLSM images of SKOV-3 cells incubated with 10 μg/mL DTPR for 4 h under the standard cell culture conditions and the nuclei were stained by Hoechst 33258. Red channel: excitation wavelength, 808 nm; emission collected: 640-740 nm. Blue channel: excitation wavelength, 720 nm; emission collected: 424-476 nm.

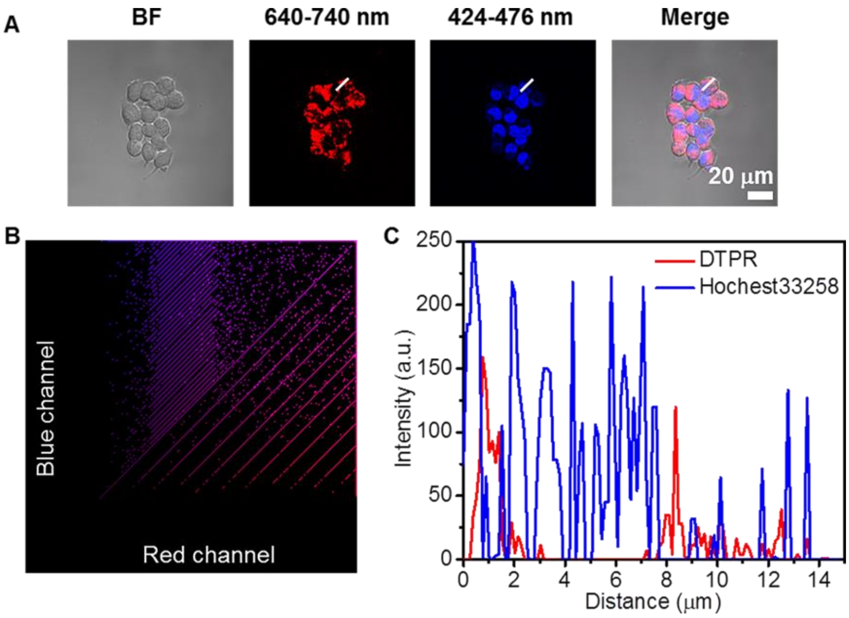

Figure S17. (A) CLSM images of SKOV-3 cells incubated with 10 μg/mL DTPR for 4 h in cell culturing condition and the nuclei were stained by Hoechst 33258. **(B)** Fluorescence intensity correlation plot, the Pearson's coefficient of DTPR and Hochest 33258 was 0.4. **(C)** Intensity profile of regions of interest (ROI) across SKOV-3 cells. Red channel: excitation wavelength, 808 nm; emission collected: 640-740 nm. Blue channel: excitation wavelength, 720 nm; emission collected: 424-476 nm. Scale bar: 20 μm.

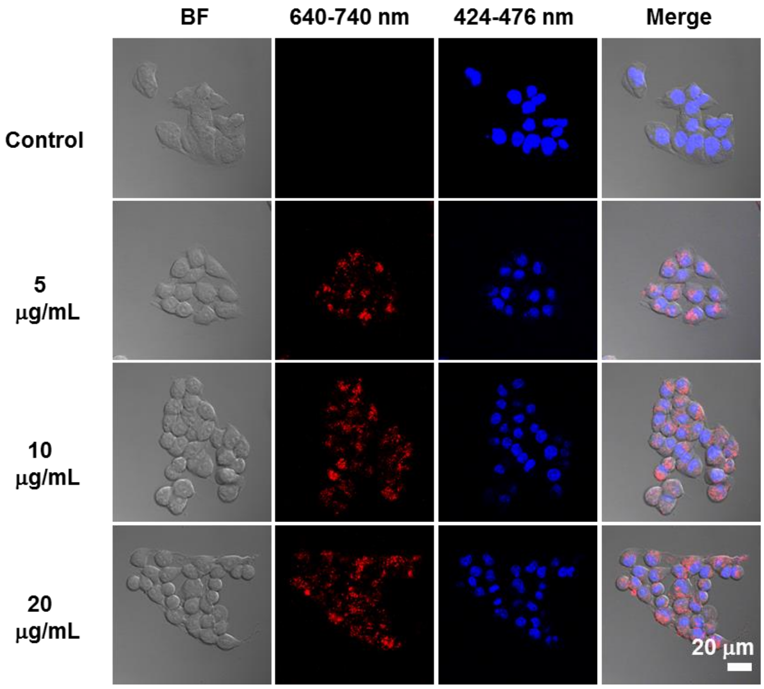

Figure S18. CLSM images of SKOV-3 cells with different concentrations of 5 μg/mL, 10 μg/mL, 20 μg/mL DTPR nanoparticles incubated for 4 h in cell culturing condition and the nuclei were stained by Hoechst 33258. Red channel: excitation wavelength, 488 nm; emission collected: 640-740 nm. Blue channel: excitation wavelength, 720 nm; emission collected: 424-476 nm. Scale bar: 20 μm.

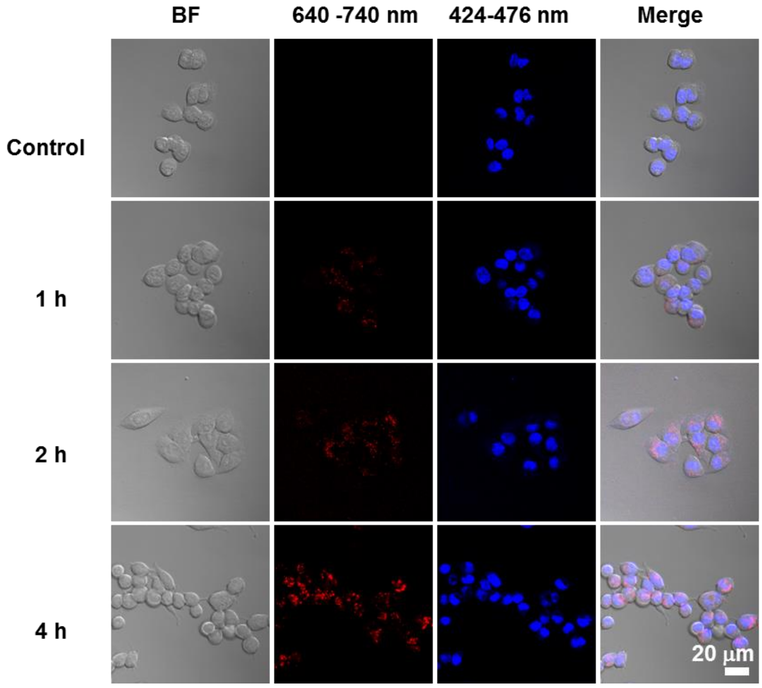

Figure S19. CLSM images of SKOV-3 cells incubated with 10 μg/mL DTPR for 1 h, 2 h, 4 h in cell culturing condition and the nuclei were stained by Hoechst 33258. Red channel: excitation wavelength, 488 nm; emission collected: 640-740 nm. Blue channel: excitation wavelength, 720 nm; emission collected: 424-476 nm. Scale bar: 20 μm.

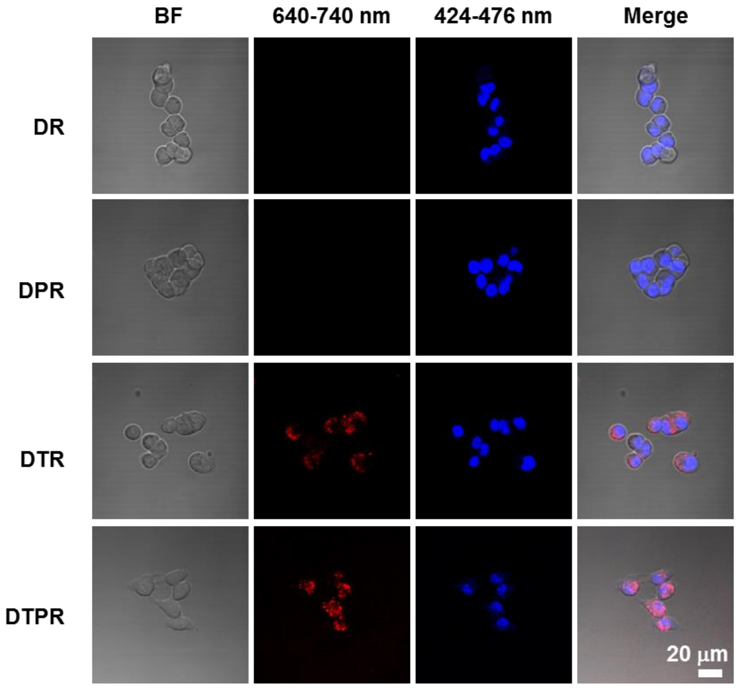

Figure S20. CLSM images of SKOV-3 cells incubated with 10 μg/mL DR, DPR, DTR and DTPR for 4 h in cell culturing condition and the nuclei were stained by Hoechst 33258. Red channel: excitation wavelength, 850 nm; 640-740 nm. Blue channel: excitation wavelength, 720 nm; emission collected: 424-476 nm. Scale bar: 20 μm.

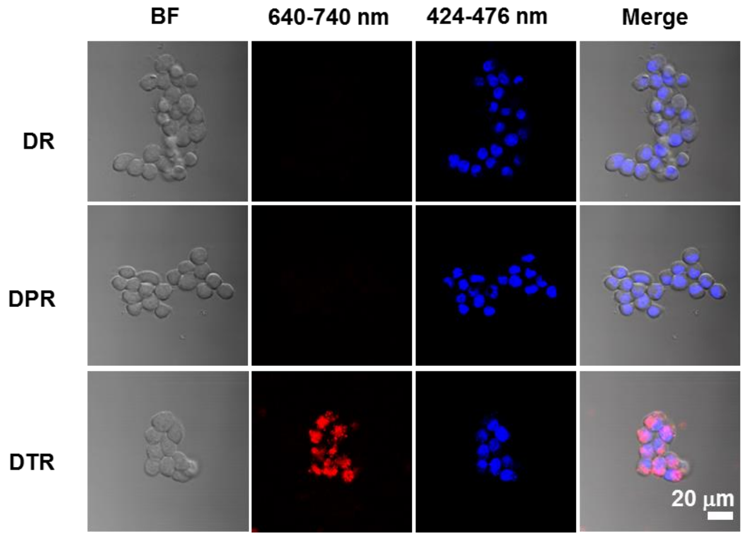

Figure S21. CLSM images of SKOV-3 cells incubated with 10 μg/mL DR, DPR and DTR for 4 h in cell culturing condition and the nuclei were stained by Hoechst 33258. Red channel: excitation wavelength, 808 nm; 640-740 nm. Blue channel: excitation wavelength, 720 nm; emission collected: 424-476 nm. Scale bar: 20 μm.

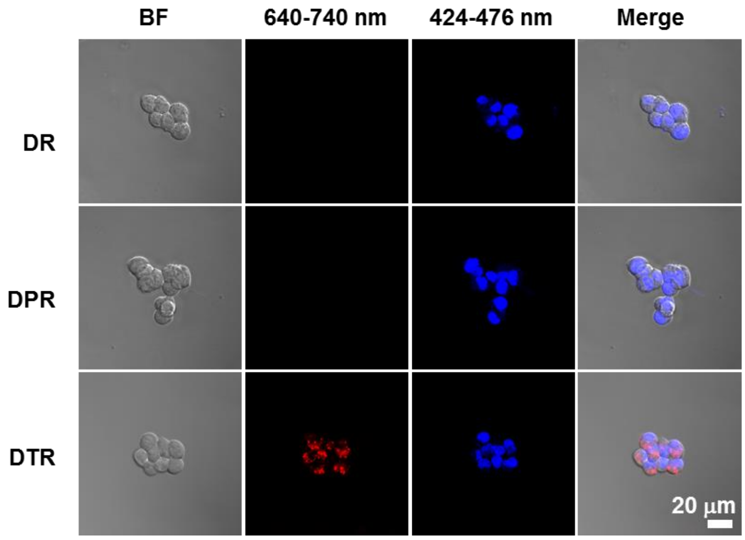

Figure S22. CLSM images of SKOV-3 cells incubated with 10 μg/mL DR, DPR and DTR for 4 h in cell culturing condition and the nuclei were stained by Hoechst 33258. Red channel: excitation wavelength, 488 nm; emission collected: 640-740 nm. Blue channel: excitation wavelength, 720 nm; emission collected: 424-476 nm. Scale bar: 20 μm.

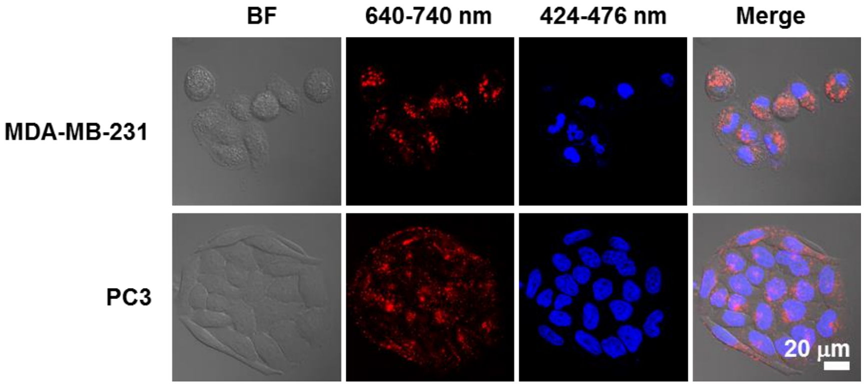

Figure S23. CLSM images of MDA-MB-231, PC3 cells incubated with 10 μg/mL DTPR for 4 h in cell culturing condition and the nuclei were stained by Hoechst 33258. Red channel: excitation wavelength, 488 nm; 640-740 nm. Blue channel: excitation wavelength, 720 nm; emission collected: 424-476 nm. Scale bar: 20 μm.

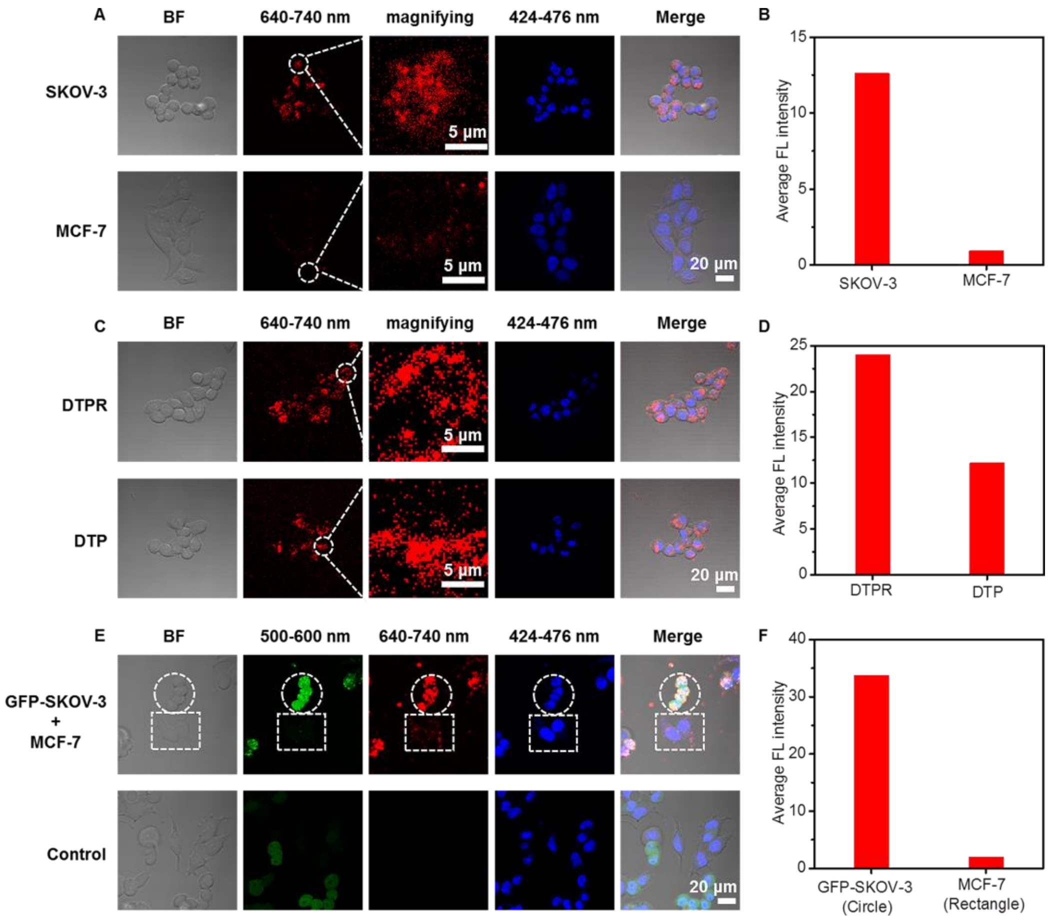

Figure S24. (A) CLSM images of SKOV-3 and MCF-7 cells after incubation with DTPR nanoparticles (10 μg/mL) for 4 h, respectively. **(B)** The corresponding average red fluorescence intensity of SKOV-3 and MCF-7 cells. **(C)** CLSM images of SKOV-3 cells after incubation with 10 μg/mL DTPR and DTP for 4 h, respectively. **(D)** The corresponding average red fluorescence intensity of SKOV-3 cells. **(E)** The CLSM images of co-cultured GFP-SKOV-3 and MCF-7 cells after incubation with 10 μg/mL DTPR for 4 h, and then 10 μM Hoechst 33258 for 30 min. **(F)** The average red fluorescence intensity of GFP-SKOV-3 (circle) and MCF-7 (rectangle) co-cultured cells incubating with DTPR. DTPR channel: excitation wavelength, 850 nm; emission collected: 640-740 nm. GFP channel: excitation wavelength, 488 nm; emission collected: 500-600 nm. Hoechst 33258 channel: excitation wavelength, 720 nm; emission collected: 424-476 nm. Scale bars: 20 μm.

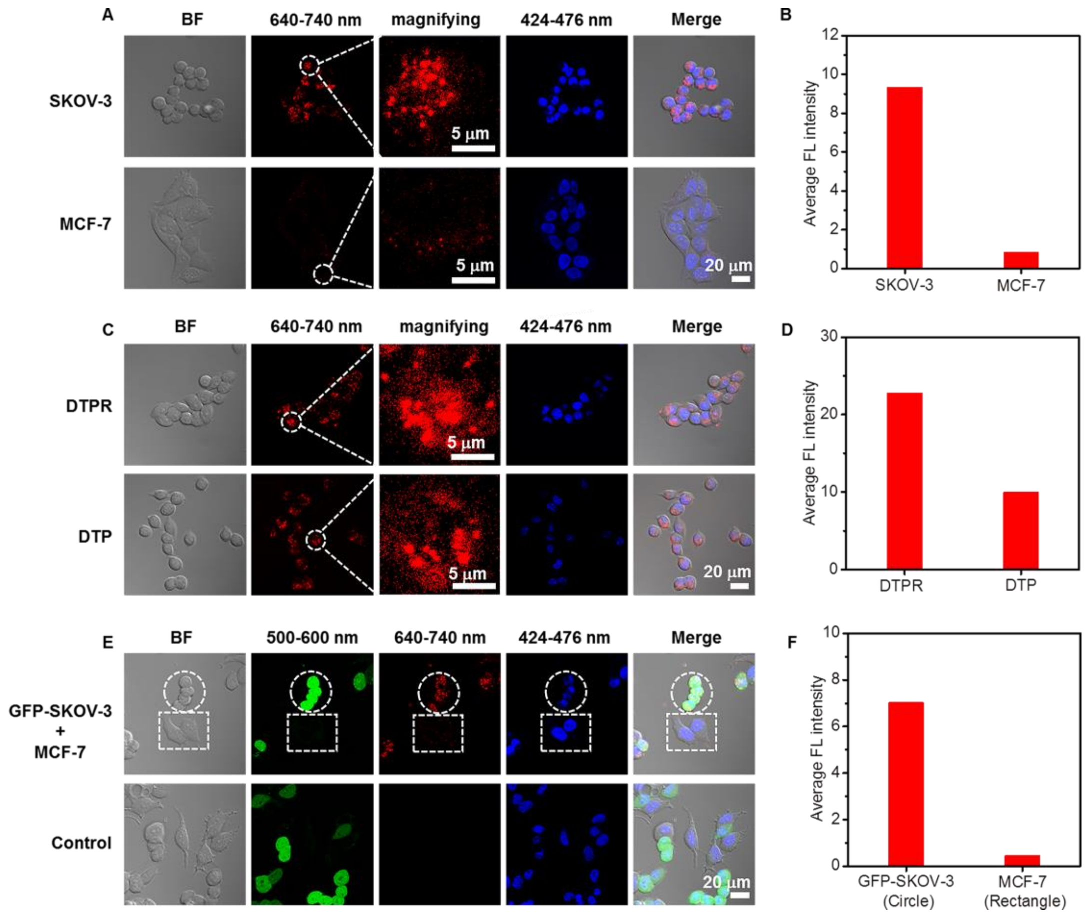

Figure S25. (A) CLSM images of SKOV-3 and MCF-7 cells after incubation with DTPR (10 μg/mL) for 4 h, respectively. **(B)** The corresponding average red fluorescence intensity of SKOV-3 and MCF-7 cells. **(C)** CLSM images of SKOV-3 cells after incubation with 10 μg/mL DTPR and DTP nanoparticles for 4 h, respectively. **(D)** The corresponding average red fluorescence intensity of SKOV-3 cells. **(E)** The CLSM images showing the GFP-SKOV-3 and MCF-7 co-cultured cells after incubated with 10 μg/mL DTPR for 4 h, and and 10 μM Hoechst 33258 was added for another 30 min incubation. **(F)** The average red fluorescence intensity of GFP-SKOV-3 (circle) and MCF-7 (rectangle) co-cultured cells incubated with DTPR. DTPR channel: excitation wavelength, 488 nm; emission collected: 640-740 nm. GFP channel: excitation wavelength, 488 nm; emission collected: 500-600 nm. Hoechst 33258 channel: excitation wavelength, 720 nm; emission collected: 424-476 nm. Scale bars: 20 μm.

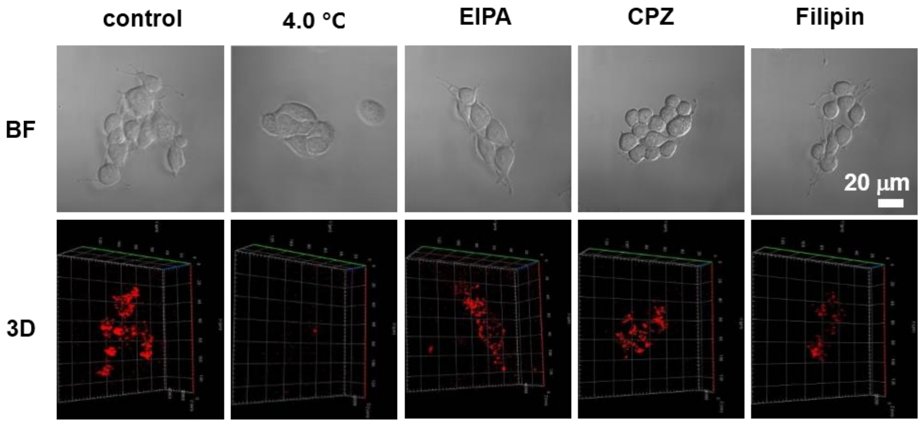

Figure S26. The bright field of CLSM and three-dimensional images of SKOV-3 cells incubated with 10 μg/mL DTPR before and after treatment with low temperature (4.0 ℃) or various inhibitors (CPZ, EIPA or filipin). Red channel: excitation wavelength, 808 nm; emission collected: 640-740 nm. Scale bar: 20 μm.

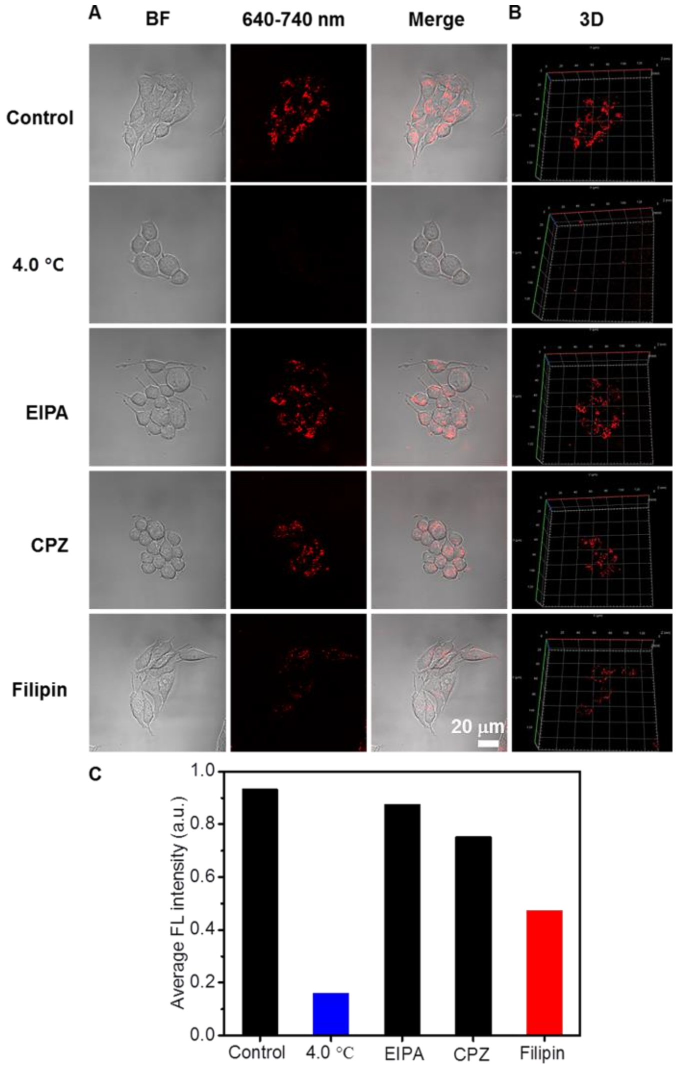

Figure S27. (A) CLSM, **(B)** three-dimensional images and **(C)** average FL intensities of SKOV-3 cells incubated with 10 μg/mL DTPR before (control) and after treatment with low temperature (4 ℃) or various inhibitors (CPZ, EIPA or filipin). Red channel: excitation wavelength, 850 nm; emission collected: 640-740 nm. Scale bar: 20 μm.

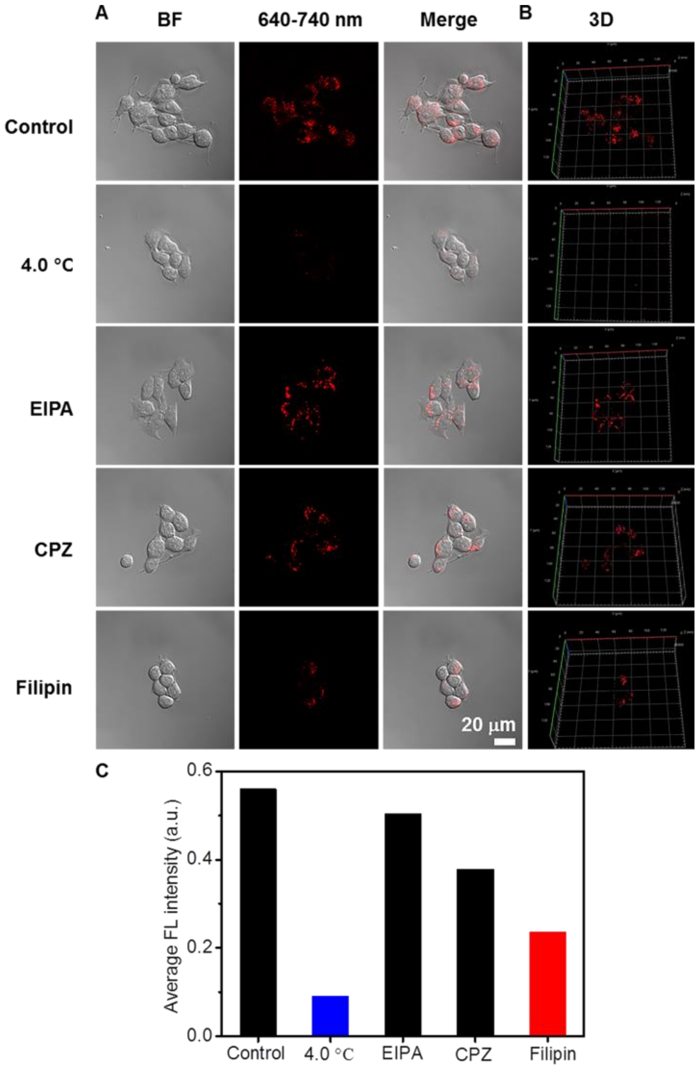

Figure S28. (A) CLSM, **(B)** three-dimensional images and **(C)** corresponding average FL intensities of SKOV-3 cells incubated with 10 μg/mL DTPR before (control) and after treatment with low temperature (4 ℃) or various inhibitors (CPZ, EIPA or filipin). Red channel: excitation wavelength, 488 nm; emission collected: 640-740 nm. Scale bar: 20 μm.

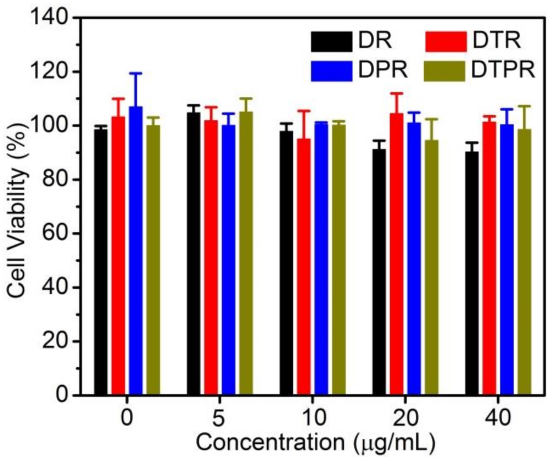

Figure S29. Viabilities of SKOV-3 cells after incubation with different concentrations of DR, DTR, DPR and DTPR nanoparticles for 24 h. All the data were presented as the average ± standard error (n = 5).

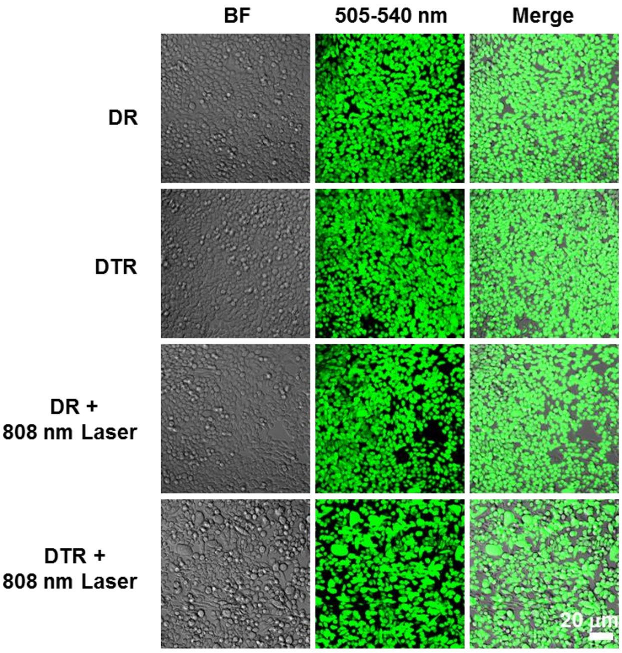

Figure S30. CLSM images of DR, DTR induced photothermal ablation after the various treatments (40 μg/mL DR only, 40 μg/mL DTR only, 40 μg/mL DR + 808 nm laser and 40 μg/mL DTR + 808 nm laser group). Green channel: excitation wavelength, 488 nm; emission collected: 505-540 nm, Scale bars: 20 μm.

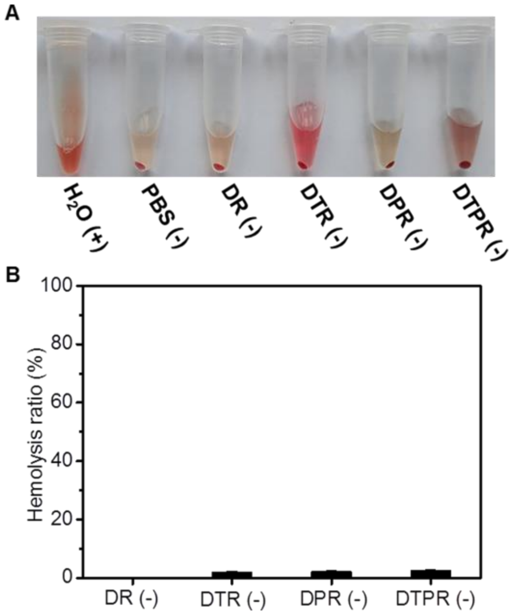

Figure S31. (A) Photographs of hemolysis assay after incubation with ultrapure water (positive control), PBS (negative control), and DR, DTR, DPR, DTPR nanoparticles for 3 h in dark. The presence of large amounts of hemoglobin in the supernatant is observed only in the positive control; **(B)** The hemolysis ratio induced by DR, DTR, DPR and DTPR nanoparticles with 100 μg/mL incubated at 37 ℃ for 3 h in dark.

Groups	Samples	Treatment
		808 nm Laser
a	PBS	-
b		+
c	DR	-
d		+
e	DTR	+
f	DPR	-
g		+
h	DTPR	-
i		+

Table S2. Different treatments among DR, DTR, DPR and DTPR nanoparticles groups.

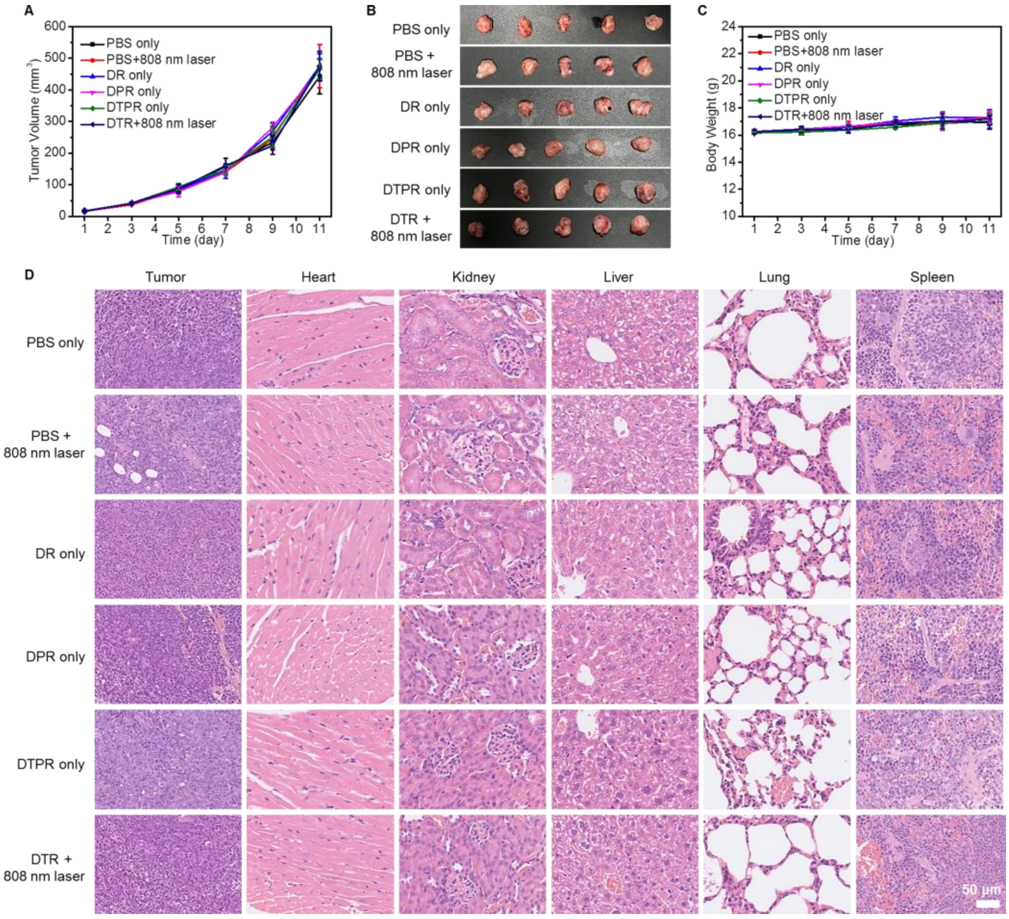

Figure S32. (A) Time-dependent tumor growth curves after different treatments (n = 5). **(B)** Photos of tumors after various treatments were taken at day 11 (n = 5). **(C)** Body weight changes of SKOV-3 tumor-bearing mice that received various treatments as indicated (n = 5). **(D)** Histopathological examinations via H&E staining of tumor, heart, kidneys, liver, lung, spleen of various treatments groups as indicated. Scale bars: 50 μm.
